# Identification of a Biosynthetic Gene Cluster for the Production of the Blue-Green Pigment Xylindein by the Fungus *Chlorociboria aeruginascens*

**DOI:** 10.1021/acs.jnatprod.4c00350

**Published:** 2025-01-23

**Authors:** Yanfang Guo, Jorge Navarro-Muñoz, Caroline Rodenbach, Elske Dwars, Chendo Dieleman, Bart van den Hout, Bazante Sanders, Miaomiao Zhou, Ayodele Arogunjo, Russell J. Cox, Arnold J. M. Driessen, Jérôme Collemare

**Affiliations:** † Fungal Natural Products Group, Westerdijk Fungal Biodiversity Institute, 3584 CT Utrecht, Netherlands; ⊥ Department of Molecular Microbiology, University of Groningen, 9747 AG Groningen, Netherlands; ‡ Bioinformatics Group, Wageningen University and Research, 6708 PB Wageningen, Netherlands; § School of Life Sciences and Technology, Avans University of Applied Sciences, 4818 AJ Breda, Netherlands; ∥ Institute for Organic Chemistry and BMWZ, Leibniz Universität Hannover, 30167, Hannover, Germany

## Abstract

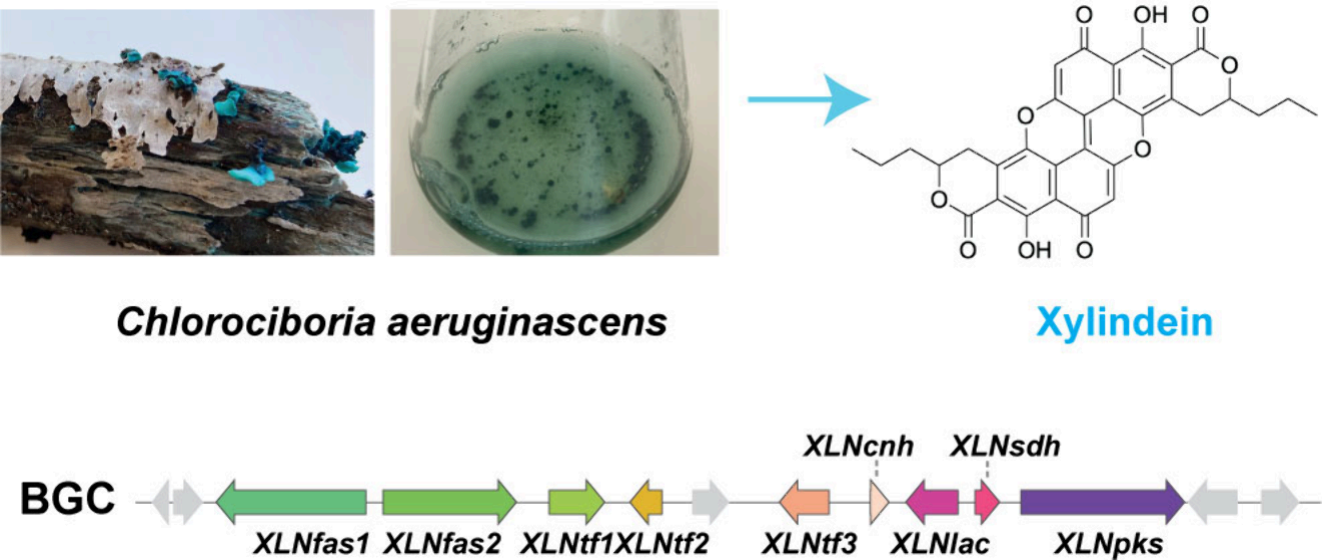

Xylindein is a blue-green pigment produced by the fungi *Chlorociboria aeruginascens* and *Chlorociboria aeruginosa.* Its stunning color and optoelectronic properties make xylindein valuable for textiles and as a natural semiconductor material. However, producing xylindein from culture broths remains challenging because of the slow growth of the *Chlorociboria* species and the poor solubility of xylindein in organic solvents. An alternative production route for obtaining pure xylindein is heterologous expression of the xylindein biosynthetic genes. Here, we resequenced the genome of *C. aeruginascens* and *C. aeruginosa*, and subsequent genome mining and phylogenetic dereplication identified a unique candidate biosynthetic gene cluster with a nonreducing polyketide synthase (nrPKS). RNA sequencing during xylindein production revealed that the core gene *XLNpks* is co-regulated with eight other genes at the locus. Among those, *XLNfas1* and *XLNfas2* encode a putative fatty acid synthase, which likely provides the starter unit to XLNpks. Attempts to heterologously express in *Aspergillus oryzae XLNpks* alone or in combination with *XLNfas1* and *XLNfas2* did not yield any intermediate, but expression of the closely related viriditoxin nrPKS (VdtA) produced the expected intermediate. Based on our results, we propose a biosynthetic route to xylindein and suggest that the obtained *A. oryzae* transformants open ways to further study xylindein biosynthesis.

Blue-green-stained wood is commonly found in humid areas of temperate forests worldwide. This color is usually due to wood colonization by the Leotiomycetes fungi *Chlorociboria aeruginascens* or *Chlorociboria aeruginosa*, which produce the blue-green pigment xylindein. This compound was one of the first isolated fungal secondary metabolites (SMs), extracted from 20 kg of stained wood from the Fontainebleau forest near Paris in 1868.^1^ The stunning color and resistance against UV light make xylindein valuable in textile coloration and the decorative wood industry. Xylindein-spalted wooden artifacts produced 500 years ago still exhibit this bright cyan color.^2^ More recently, xylindein has been investigated for its optoelectronic performance as a natural semiconductor material.^3^ Because of these properties, production of xylindein for industrial applications has been investigated.^4,5^ Xylindein cannot be easily produced by chemical synthesis,^6^ and its extraction and purification from culture broths are cumbersome and inefficient due to the slow growth of *Chlorociboria* species and its poor solubility in common organic solvents.^7^

Heterologous expression has been widely used to elucidate biosynthetic pathways of fungal SMs,^8^ but it is also a promising alternative strategy for the production of valuable metabolites from fungi that are difficult to manipulate such as *C. aeruginascens*.^9^ For example, titers up to 1.3 g L^–1^ of the hybrid cyclodepsipeptide hexa-bassianolide were produced in an engineered *Aspergillus niger* strain.^10^ A seven-gene cluster for producing the antibiotic pleuromutilin was heterologously expressed in *Aspergillus oryzae* and gave a significant increase in production by more than 3 orders of magnitude, while no improvement in yield was achieved by using various targeted approaches in the natural basidiomycete producer *Clitopilus passeckerianus*.^11^ Heterologous expression in *A. oryzae* also benefits from fast growth under controlled laboratory conditions, genetic tractability for further engineering, and mycotoxin-free production.^12^

Xylindein is a dimeric naphtho-α-pyranone polyketide that resembles talaroderxine A produced by *Talaromyces derxii* (Figure 1).^13^ The production of xylindein is expected to involve a nonreducing polyketide synthase (nrPKS).^14,15^ Several fungal nrPKSs have been reported to produce similar naphtho-α-pyranone intermediates, including VdtA and Ctb1, which are involved in the biosynthesis of viriditoxin and cercosporin, respectively (Figure 1).^16−18^ The chemical structures of xylindein and viriditoxin monomers share similarities that suggest common biosynthetic steps. The viriditoxin biosynthetic pathway from the fungus *Paecilomyces variotii* was elucidated using targeted gene deletion and heterologous expression in *Aspergillus nidulans*.^16,17^ The nrPKS VdtA produces the pyranone backbone, which is methylated by the *O*-methyltransferase VdtC and reduced by the short-chain dehydrogenase VdtF.^17^ The phenol-coupling dimerization of viriditoxin involves the laccase VdtB and the catalytically inactive hydrolase VdtD.^17^ Homologous biosynthetic gene clusters (BGCs) to viriditoxin were also reported in diverse fungal species known to produce naphthopyrones like vioxanthin and xanthoepocin.^19^ Although the nrPKSs found in these BGCs were not characterized, the laccase Av-VirL found in the *Aspergillus viridinutans* predicted naphtho-α-pyranone BGC exhibited the same phenol-coupling activity as VdtB.^19^

**Figure 1:**
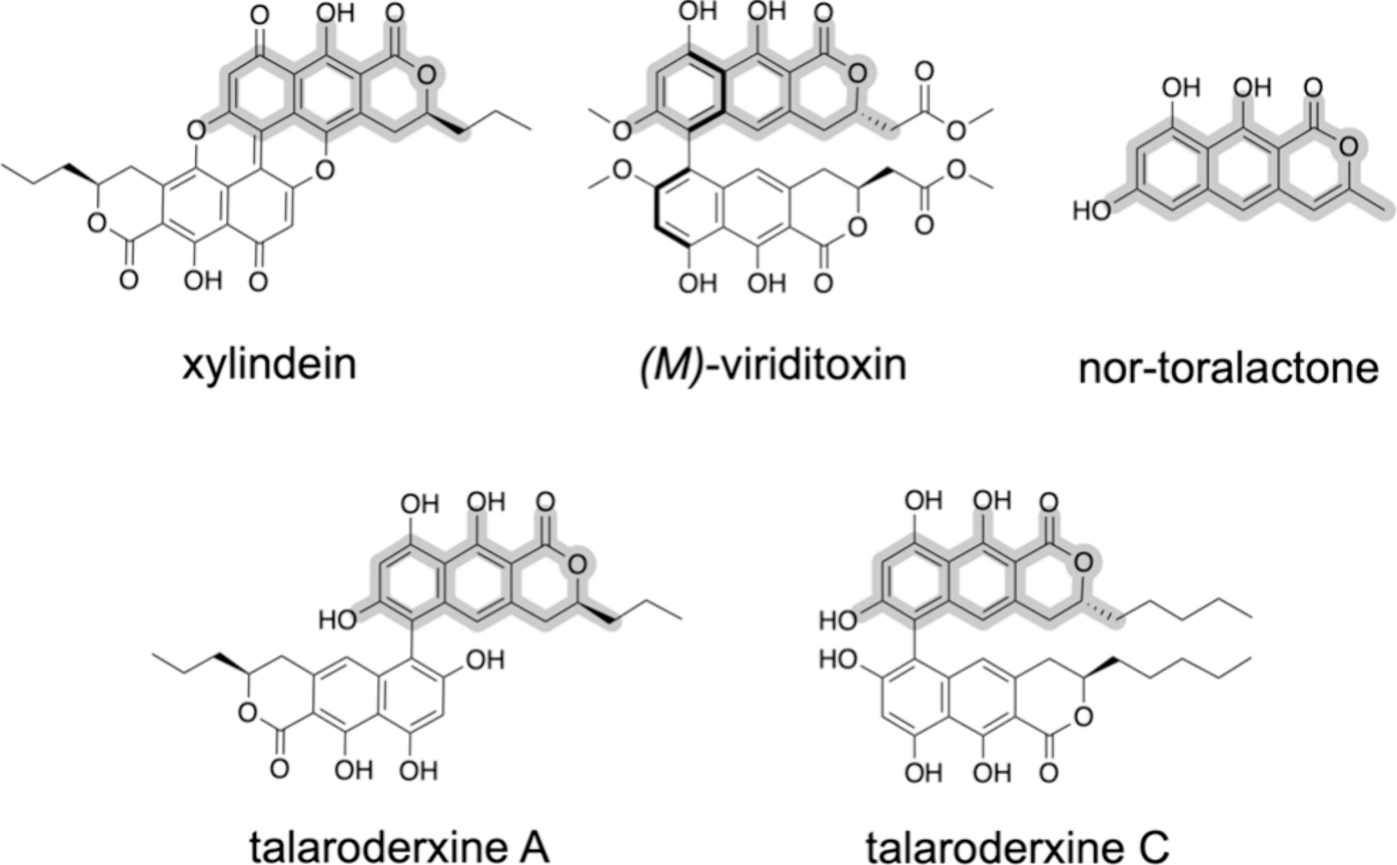
Xylindein and related compounds. The common naphtho-α-pyranone backbone is highlighted in gray. Nor-toralactone is a biosynthetic precursor of cercosporin.

The genome sequence of *C. aeruginasens* was recently reported,^14^ and its analysis indicated the presence of 32 BGCs, including 14 for the production of polyketide compounds. Yet, no BGC has been linked to xylindein. In this study, we resequenced the genome of *C. aeruginascens*, as well as of the related *C. aeruginosa* species, and performed genome mining and phylogenetic dereplication to identify a candidate BGC for the production of xylindein. We initiated the elucidation of this biosynthetic pathway using heterologous expression in *Aspergillus oryzae*.

## Results and Discussion

### Phylogenetic Dereplication of *C. aeruginascens* Identifies a Single Candidate nrPKS for Xylindein Production

Linking biosynthetic genes to an already characterized molecule can be done by exploring the genome of the fungal producer, based on hypotheses according to the predicted enzymatic requirements to yield the chemical structure of interest. Retro-biosynthesis of xylindein indicates that the monomeric precursor released by the nrPKS is likely related to viriditoxin monomer and nor-toralactone, the precursor of cercosporin (Figure 1). Based on this observation, the nrPKS controlling xylindein production is likely related to the corresponding VdtA and Ctb1 enzymes, which both belong to group IV of nrPKSs.^20^

We thus embarked on mining the *C. aeruginascens* genome, searching for group IV nrPKS genes. The available genome assembly of *C. aeruginasens* DSM 107184 is highly fragmented, with 588 contigs in total.^14^ To obtain a better assembly for genome mining, we sequenced the *C. aeruginascens* CBS 122017 strain, as well as two *C. aeruginosa* strains, using Oxford Nanopore long-read technology. The newly obtained assembly of *C. aeruginascens* is slightly larger with a size of 38.3 Mb in a total of 13 assembled contigs and with 9457 predicted genes (Table S1). The assembly of *C. aeruginosa* CBS 139.28 is similar in size (38.9 Mb) and has a similar number of predicted genes (9630). In contrast, the assembly of *C. aeruginosa* CBS 123.57 is larger, with a size of 45.9 Mb, and encodes more predicted genes (12,210, Table S1). The low number of large contigs over 1 Mb in the *C. aeruginascens* assembly and detection of telomeric repeats in all three assemblies suggest that *Chlorociboria* species comprise between four and six chromosomes (Figure S1). We identified regions with predicted BGCs using antiSMASH (Table S2).^21^ Out of the 14 predicted PKSs in *C. aeruginascens*, only two share the conserved domain organization of nrPKSs, which are encoded by *g4260* and *g423* genes, respectively (Table S3).

Similarly, the *C. aeruginosa* CBS 139.28 assembly contains only two nrPKS genes, while the *C. aeruginosa* CBS 123.57 assembly encodes five of them (Table S2 and Table S3).

**Figure 2:**
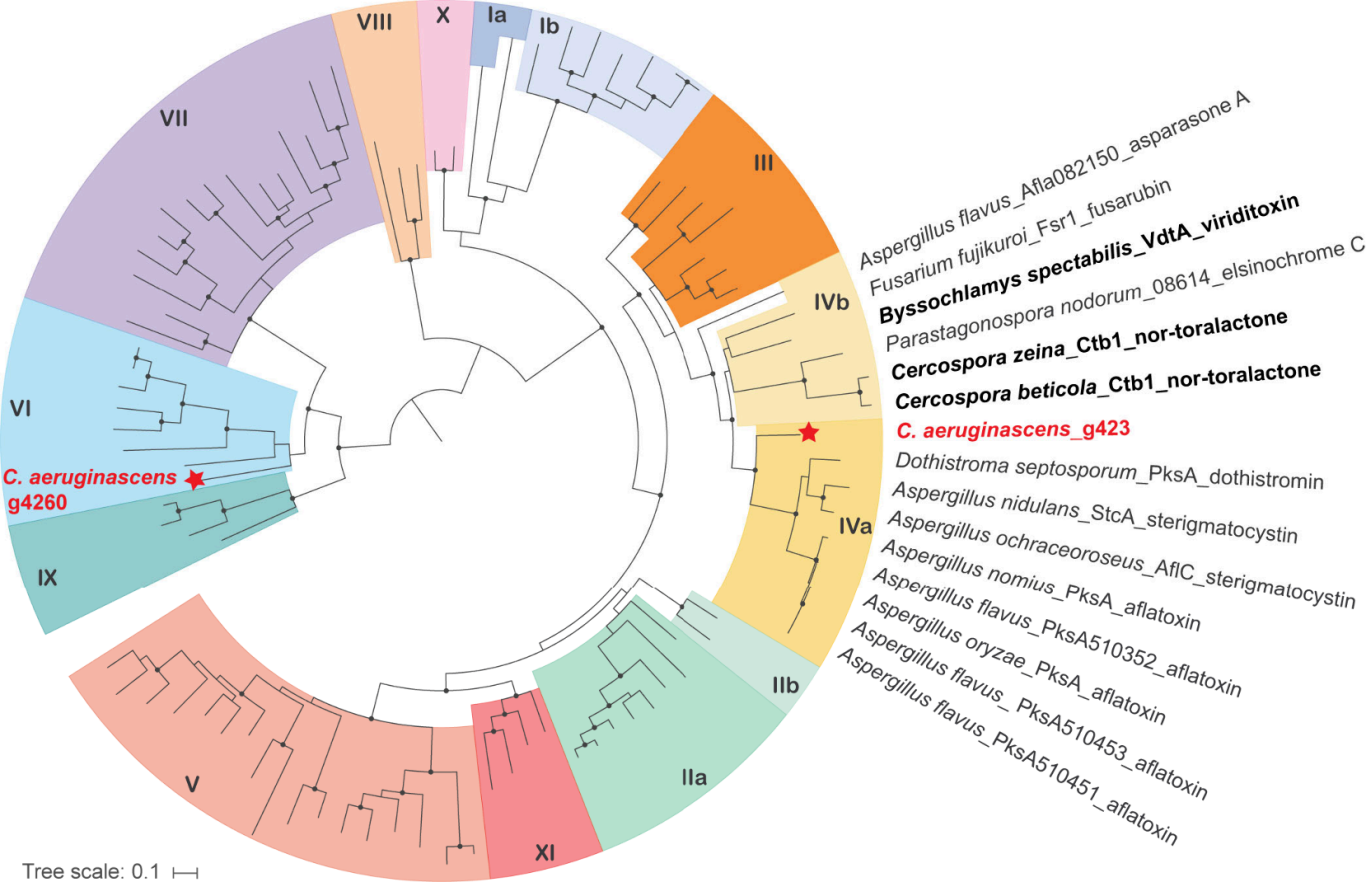
Phylogenetic dereplication identifies a candidate nonreducing polyketide synthase (nrPKS) for the production of xylindein. A maximum likelihood phylogenetic tree was built with characterized nrPKSs from the MIBiG database and literature. Ultrafast bootstrap values over 95 and likelihood ratio tests above 80 are indicated with black dots at the nodes. The tree is midpoint rooted. Colors and numbers indicate the nrPKS phylogenetic clades.

To determine if one of the two nrPKSs encoded in the *C. aeruginascens* genome is related to group IV nrPKSs, we performed a phylogenetic dereplication that included 92 characterized nrPKSs from the Minimum Information about a Biosynthetic Gene Cluster (MIBiG) database and from the literature^20^ (Supplementary files S1, S2, and S3). The obtained maximum likelihood phylogenetic tree showed that the nrPKS encoded by *g4260* belongs to group VI, whose PKSs produce methylorsellinic acid or 3,5-dimethylorsellinic acid (Figure 2).^20^ These molecules are tetraketides derived from an acetate starter unit, which is not consistent with the structure of xylindein that is likely to be a heptaketide derived from a C_4_ starter unit. In contrast, the other nrPKS encoded by *g423* belongs to the expected group IV, together with the nrPKSs involved in the biosynthesis of fusarubin, viriditoxin, cercosporin, and aflatoxins with a strong branch support (Figure 2). However, g423 and VdtA do not form a monophyletic clade. Instead, g423 forms a strongly supported outgroup to the dothistromin, sterigmatocystin, and aflatoxin nrPKSs (Figure 2). These latter enzymes were previously separated into group IVa, consistent with the production of an anthraquinone intermediate rather than a pyranone backbone.^20^

The phylogenetic tree presented here confirms that the ancestor of group IV nrPKSs likely released a naphthopyrone intermediate. The divergence of group IVa is likely linked to the modification of the starter unit, as nrPKSs from this group use hexanoyl-CoA produced by fatty acid synthases encoded in the BGC instead of acetyl-CoA as the polyketide starter unit.^22^ Phylogenetic dereplication with all nrPKSs encoded in the four *Chlorociboria* assemblies included in this study confirms that g423 is the only nrPKS conserved in all four strains (Figure S2; Supplementary files S4, S5, and S6). Altogether, the phylogenetic dereplication identified a single candidate nrPKS for xylindein production, which we hereafter name XLNpks.

### Prediction of a Unique Biosynthetic Gene Cluster for the Production of Xylindein

**Figure 3:**
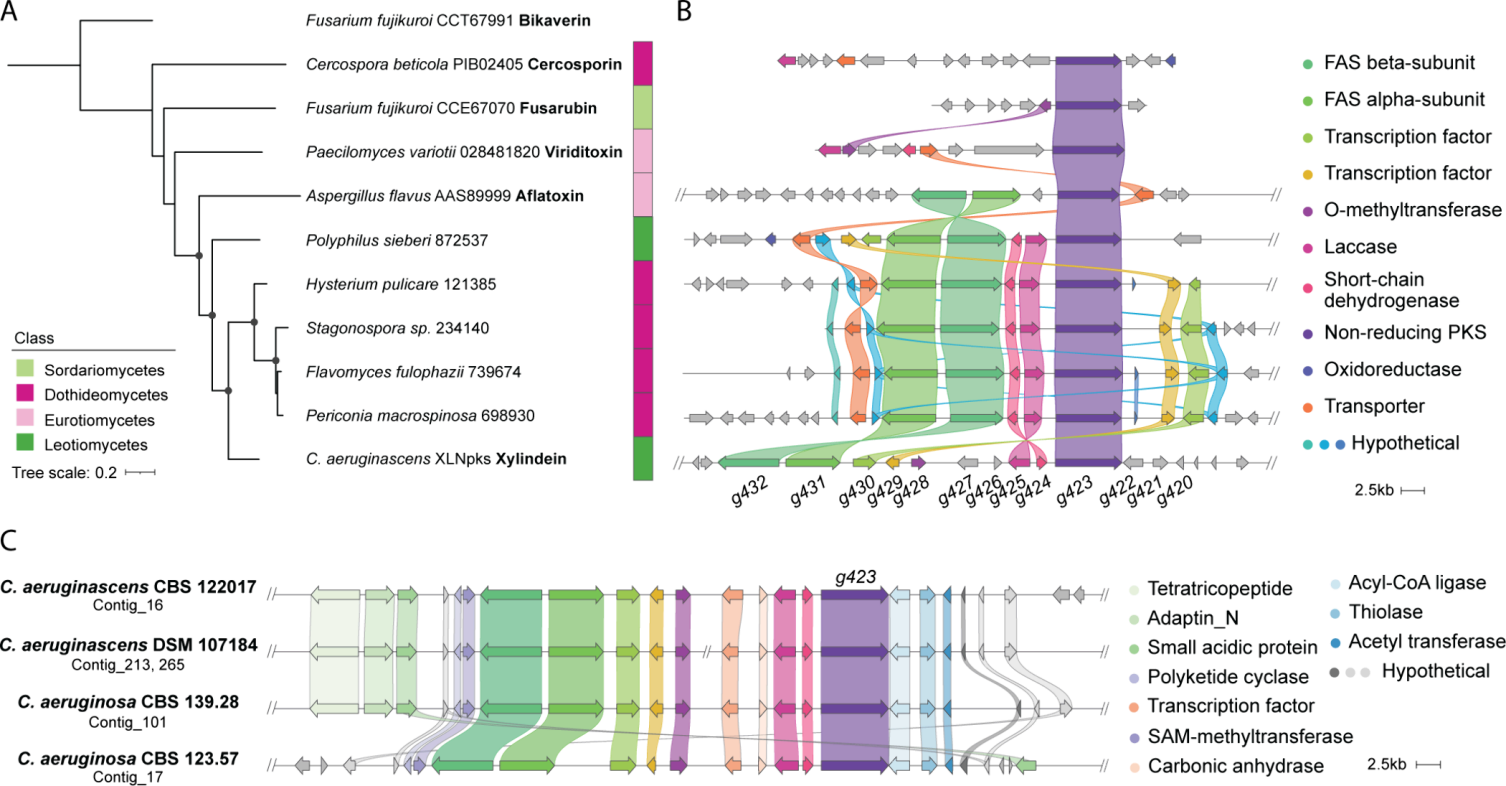
A predicted biosynthetic gene cluster (BGC) for the production of xylindein and related compounds. (A) Maximum-likelihood phylogenetic tree of XLNpks homologues and characterized nrPKSs. The bikaverin nrPKS from *Fusarium fujikuroi* was used as an outgroup. Ultrafast bootstrap values over 95 and a likelihood ratio test above 80 are indicated with black dots at the nodes. Protein identifiers from JGI Mycocosm or GenBank accession numbers are indicated next to the species name. (B) Loci of the nrPKS genes as predicted by antiSMASH were compared using Clinker. (C) *XLNpks* loci in the *Chlorociboria* genus were compared using Clinker. Homologous genes are represented by the same colored arrows with connections.

Although xylindein is a unique pigment, so far only reported to have been isolated from the *Chlorociboria* genus, we searched for orthologs of *XLNpks* in publicly available fungal genomes. This search identified only four orthologous nrPKSs from distant Dothideomycetes species and one close homologue in the Leotiomycetes *Polyphilus sieberi* (Figure 3A). While there is no report of naphtho-α-pyranones produced by these Dothideomycetes species, *P. sieberi* was recently found to produce talaroderxine C, a compound closely related to xylindein and talaroderxine A, but exhibiting a hexaketide starter unit.^13^ This phylogenetic analysis including close homologues confirms that the XLNpks share a common ancestry with the aflatoxin nrPKS (Figure 3A), which diverged from a common ancestor with other naphtho-α-pyranone-producing nrPKSs like VdtA and Ctb1.

To determine a potential BGC for xylindein production, we then compared the *XLNpks* genomic locus to those of all its close homologues (Figure 3B; Table S4; Table S5). The gene components at the nrPKS loci in the different species are consistent with the phylogenetic relationships. A clear conserved BGC is found within the XLNpks monophyletic clade as well as in *P. sieberi*, which consists of the nrPKS gene and a common set of six genes that encode putative tailoring enzymes or transcription factors based on their functional conserved domains (Figure 3B; Table 1). *XLNtf1* (*g430*) and *XLNtf2* (*g429*) encode transcription factors, with the latter being related to the aflatoxin *aflR* regulator (Table 1), which might be involved in the regulation of these BGCs. Reminiscent of the aflatoxin BGC, two genes encode a pair of fatty acid synthase (FAS) subunits, *XLNfas1* (*g432*) and *XLNfas2* (*g431*). The presence of this pair of genes is consistent with the biosynthesis of a fatty acyl CoA starter unit (Figure 1).

**Table 1:** Functions Encoded at the *XLNpks* Locus^a^

Gene ID	Gene name	Predicted protein function	Pfam domain, E-value
g434	-	Polyketide cyclase	pfam03364, 1.02e-12
g433	-	SAM-methyltransferase	pfam13649, 3.75e-19
g432	*XLNfas1*	Fatty acid synthase beta-subunit	FAS_N pfam17828, 3.88e-07
			SAT pfam16073, 1.11e-25
			ER pfam08354, 6.36e-170
			β-meander pfam17951, 2.91e-26
			DH_N pfam13452, 3.37e-12
			DH pfam01575, 2.03e-32
			AT pfam00698, 4.59e-45
g431	*XLNfas2*	Fatty acid synthase alpha-subunit	ACP pfam18325, 6.68e-77
			FAS_I_H pfam18314, 7.40e-50
			ADH pfam00106, 8.98e-09
			KS_N pfam00109, 1.22e-11
			KS_C pfam02801, 7.83e-09
			PPT pfam01648, 4.02e-11
g430	*XLNtf1*	Transcription factor	pfam04082, 2.31e-12
g429	*XLNtf2*	Transcription factor	pfam08493, 5.25e-27
g428	-	O-methyltransferase	pfam00891, 2.23e-03
g427	*XLNtf3*	Transcription factor	pfam11951, 4.72e-05
g426	*XLNcnh*	Carbonic anhydrase	pfam00484, 2.37e-12
g425	*XLNlac*	Laccase	pfam07732, 4.93e-37
g424	*XLNsdh*	Short-chain dehydrogenase	pfam00106, 9.22e-23
			pfam13561, 2.09e-27
g423	*XLNpks*	Polyketide synthase	SAT pfam16073, 5.24e-74
			KS pfam00109, 1.54e-91
			KS_C pfam02801, 5.62e-34
			AcT pfam00698, 1.54e-40
			PT TIGR04532, 5.8e-96
			PP-b pfam00550, 7.78e-11
			TE_N pfam00108, 4.43e-05
g422	-	Acyl-CoA ligase	pfam00501, 2.84e-50
g421	-	Thiolase	pfam02803, 1.86e-52

a Only genes predicted to belong to the xylindein biosynthetic gene cluster were given a *XLN* name.

The other two conserved genes, *XLNsdh* (*g424*) and *XLNlac* (*g425*), encode a short-chain dehydrogenase and a laccase, respectively (Figure 3B; Table 1), consistent with the redox processes required for the formation of xylindein. Homologues of those genes are found in the viriditoxin BGC (Figure 3B), reflecting similar chemical steps involved in both pathways.

The *XLNpks* locus also contains an *O*-methyltransferase gene (*g428*) that is found in the viriditoxin BGC only (Figure 3B; Table S4; Table S5). However, in contrast to viriditoxin, no hydroxy group is methylated in the xylindein structure (Figure 1), and this gene is unlikely to be involved in the biosynthetic pathway. In addition, the *XLNpks* locus in *C. aeruginascens* contains two genes in between *XLNtf2* and *XLNlac* which encode a third transcription factor (*g427*; *XLNtf3*) and a putative carbonic anhydrase (*g426*; *XLNcnh*, Figure 3B; Table 1). No close homologue of *XLNcnh* is found in any of the other species, and *XLNtf3* is only found in the *P. sieberi* genome but on a different scaffold (Table S4).

Downstream of *XLNpks* are three genes that encode enzymes commonly found in secondary metabolite biosynthetic pathways: an acyl-CoA ligase (*g422*); a thiolase (*g421*); and an acetyl transferase (*g420*). These genes are not conserved at the locus in other species included in this study (Figure 3B). In particular, no close homologue to *g422* is found in the four Dothideomycetes species (Table S4). The two genes upstream of the FAS genes encode a polyketide cyclase (*g434*) and a SAM-methyltransferase (*g433*) that are not conserved at the locus in other species included in this study (Figure 3B; Table S4) and thus are unlikely to belong to the BGC.

Comparison of the locus within the *Chlorociboria* genus shows that the predicted BGC, from two FAS genes to the nrPKS gene, is fully conserved in all four strains (Figure 3C; Table S5). Although xylindein shares structural similarities with viriditoxin, the xylindein BGC shares homologies not only with the viriditoxin BGC but also with the aflatoxin BGC (Figure 3B). Because XLNpks share common ancestry with the aflatoxin nrPKS, the recruitment of the FAS genes likely occurred in the common ancestor of these BGCs.

### Co-regulation of Genes at the *XLNpks* Locus during Xylindein Production Defines the Putative Biosynthetic Gene Cluster

**Figure 4:**
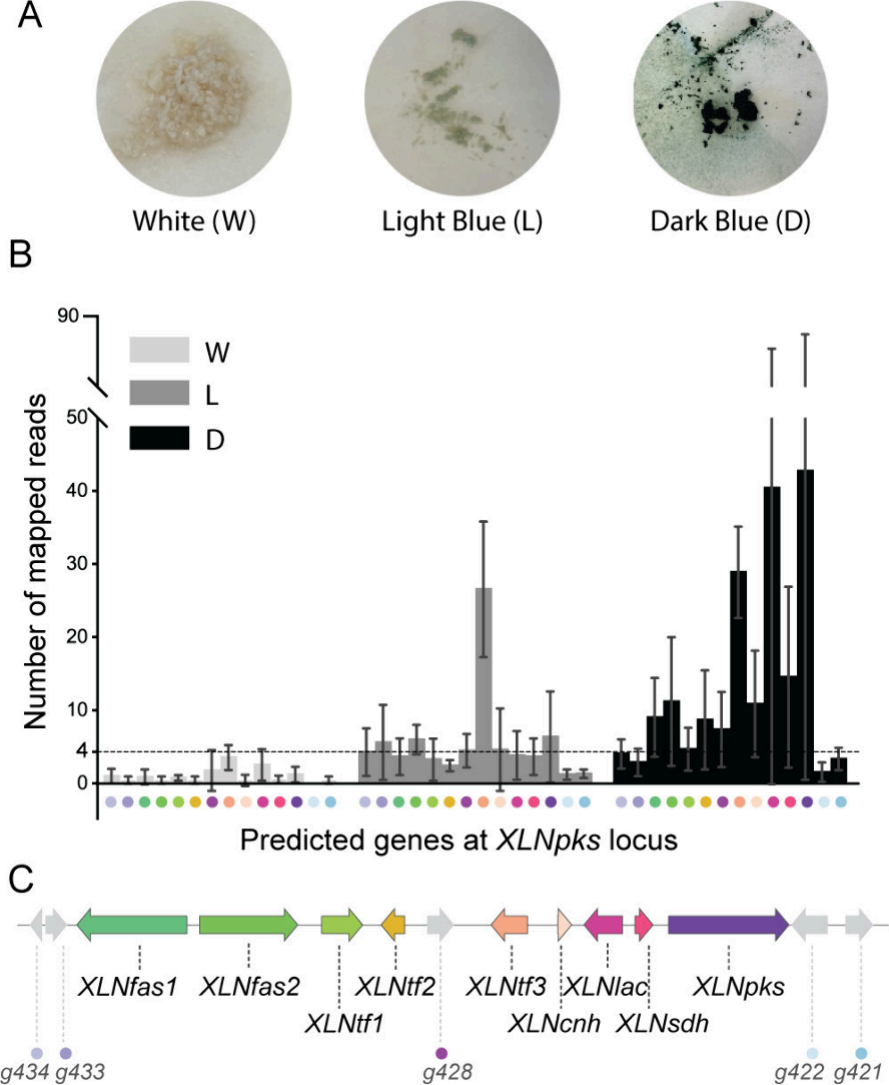
Expression of *XLNpks* locus genes during xylindein production. (A) A growth time course of *Chlorociboria aeruginascens* from white mycelium to light blue and dark blue pigmentation. (B) Expression of predicted genes at the *XLNpks* locus in *C. aeruginascens* grown under a time course, as determined by RNA sequencing. The dotted line indicates the highest expression observed for genes downstream of *XLNpks* and upstream of *XLNfas1*. Error bars represent the standard deviation of three biological replicates. Spots with different colors under the *x*-axis represent the genes at the (C) the putative xylindein BGC locus with corresponding colors.

To clearly link the candidate BCG to xylindein production, we investigated the expression of genes at the *XLNpks* locus under a growth time course from white mycelium to light blue and dark blue pigmentation (Figure 4A), corresponding to a time course of xylindein production. *C. aeruginascens* growth and xylindein production show high variability between biological replicates, which resulted in high standard deviations. Yet, this variability is found across all genes, making the comparison of their average gene expression possible. *XLNtf3* was strongly upregulated at the light blue stage and remained highly expressed at the dark blue time point (Figure 4B; Supplementary file S7). This observation suggests that XLNtf3 is likely the major regulator that activates the expression of other genes at the locus. The two other transcription factors encoding genes, *XLNtf1* and *XLNtf2*, were upregulated, especially at the dark blue stage, but they exhibited a relatively much lower expression level compared to *XLNtf3* (Figure 4B). *XLNfas1*, *XLNfas2*, and *XLNpks* were also slightly upregulated at the light blue stage and showed the highest expression level at the dark blue stage (Figure 4B). Especially at this stage, *XLNpks* is the highest expressed gene at the locus. Similarly, a high expression level of *XLNlac*, *XLNsdh*, and *XLNcnh* at the dark blue stage was observed (Figure 4B). The *O*-methyltransferase gene *g428* surprisingly exhibited a slight upregulation similar to *XLNtf1* and *XLNtf2* (Figure 4B). The genes downstream of *XLNpks* and upstream of *XLNfas1* exhibited a slight upregulation at the light blue stage, but remained weakly expressed at the dark blue stage (Figure 4B). The overall comparison indicates that the xylindein BGC is delimited by *XLNpks* and *XLNfas1*, and it seems that *XLNtf1*, *XLNtf2*, and *g428* are unlikely to be involved in xylindein production given their low expression levels and, in the *g428* case, the absence of a methylated hydroxy group.

### Heterologous Expression of Xylindein Biosynthetic Genes in *Aspergillus oryzae*

While most nrPKSs use acetyl-CoA as a starter unit,^15^ nrPKSs involved in the biosynthesis of aflatoxins and related compounds, like dothistromin, specifically use hexanoyl-CoA synthesized by FASs encoded in the BGC.^17^ Given the length of the side carbon chain in the xylindein structure and expected similarity with the viriditoxin monomer, we hypothesized that XLNfas1 and XLNfas2 evolved to provide butanoyl-CoA instead of acetyl-CoA as the starter unit to XLNpks. To characterize the early steps of xylindein biosynthesis, we thus heterologously expressed *XLNpks*, *XLNfas1*, and *XLNfas2* genes by randomly inserting them in the *A. oryzae* NSAR1 genome. The three genes were amplified from cDNA to ensure the correct removal of intron sequences.

**Figure 5:**
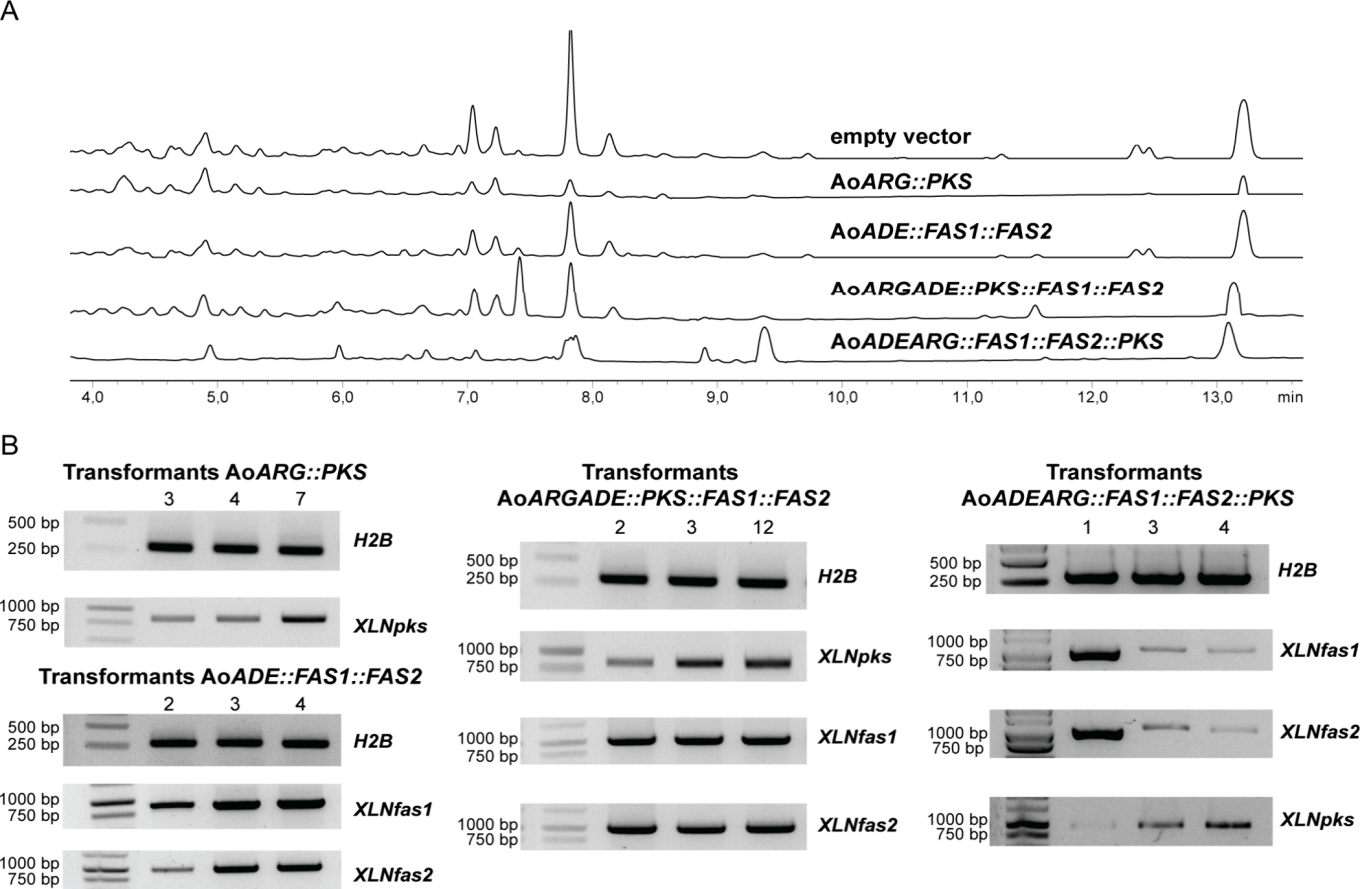
Heterologous expression of xylindein candidate biosynthetic genes. (A) Organic extracts of 4-day-old *A. oryzae* transformants carrying an empty vector and expressing candidate genes were analyzed using HPLC with a diode array detector (DAD, 190–600 nm). No intermediates were detected from all of the transformants. (B) Gene expression of the selected transformants as determined by RT-PCR. The H2B housekeeping gene was used as an expression control.

First, we generated transformants that express either *XLNpks* alone or both *XLNfas1* and *XLNfas2* (Figure 5A). Out of 11 *AoARG::PKS* transformants, seven showed *XLNpks* expression, but no intermediate was detected by HPLC (Figure 5; Table S6). This result is consistent with the requirement of XNLfas1 and XLNfas2 to provide the starter unit. Out of 10 *AoADE::FAS1::FAS2* transformants, six exhibited expressions of both the *XNLfas1* and *XLNfas2* genes (Figure 5B; Table S6).

Next, we co-expressed all three *XLNpks, XLNfas1*, and *XLNfas2* genes. For this purpose, either *XLNpks* was introduced in *AoADE::FAS1::FAS2* transformants or the *XLNfas1* and *XLNfas2* gene pair was introduced in *AoARG::PKS* transformants. In the latter case, three different *AoARG::PKS* transformants that expressed *XLNpks* were used to achieve co-expression with *XLNfas1* and *XLNfas2*. In total, 49 *AoADEARG::FAS1::FAS2::PKS* transformants were obtained, and four of 15 screened transformants exhibited expression of all three genes (Figure 5B; Table S6). Thirty-five *AoARGADE::PKS::FAS1::FAS2* transformants were collected, and four out of 12 of them exhibited expression of all three genes (Figure 5B; Table S6). However, none of these eight transformants produced any new product (Figure 5A; Table S6). To rule out the possibility of a technical issue and to test the hypothesis that pyranone nrPKSs can be functional in *A. oryzae*, we attempted the heterologous expression of the homologous nrPKS VdtA.

### Heterologous Expression of the Viriditoxin nrpks *vdtA* Gene

**Figure 6:**
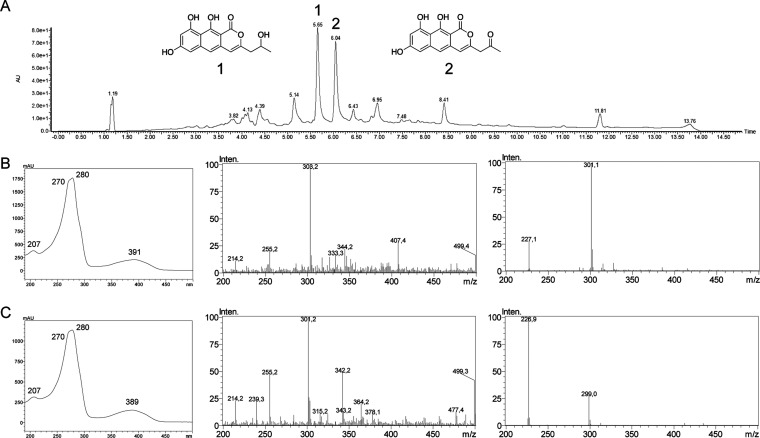
Heterologous expression of *vdtA* in*Aspergillus oryzae*. (A) HPLC analysis of the expression of *vdtA* in *A. oryzae* by diode array detector (DAD, 190–600 nm). (B) From left to right are the UV, ES^+^, and ES^–^ spectra of product 1. (C) From left to right are the UV, ES^+^, and ES^–^ spectra of product 2.

The nrPKS *vdtA* gene from *P. variotii* was previously successfully expressed in *Aspergillus nidulans*.^17^ This nrPKS is thus a good control to evaluate the ability of *A. oryzae* to produce pyranone polyketides. The *P. variotii vdtA* gene was cloned from genomic DNA and introduced into *A. oryzae* NSAR1. Fourteen out of 30 *AoADE::vdtA* tansformants produced a yellow pigment that diffused in the agar of the induction plates (Table S6). HPLC profiling of 14 transformants showed that they all produced two additional compounds that were not observed in the control (Figure 6A; Figure S3). Product 1 [retention time (*t*_R_) = 5.65 min; UV maximum = 270, 280, and 391 nm; *m*/*z* (electrospray; ES^–^) 301 [M – H]^−^] and product 2 [*t*_R_ = 6.04 min; UV maximum = 270, 280, and 389 nm; *m*/*z* (electrospray; ES^–^) 299 [M – H]^−^] are related based on their UV spectra. Product 2 was identified as 7,9,10-trihydroxy-3-(2-oxopropyl)-1*H*-benzo[*g*]isochromen-1-one, the product of VdtA in *A. nidulans*, based on UV and MS data (Figure 6).^17^ High-resolution mass spectrometry (HRMS) determined the exact mass [*m*/*z* (ES^+^) = 301.2 [M + H]^+^] and confirmed the identity of product 2 (Figure 6B; Figure S4). UV, HRMS and ^1^H NMR data of product 1 [*m*/*z* (ES^+^) 303.2 [M + H]^+^] indicates it is 7,9,10-trihydroxy-3-(2-hydroxypropyl)-1*H*-benzo[g]isochromen-1-one, a reduced derivative of product 2 (Figure 6C; Figures S4 and S5).^17^ Thus, product 1 is likely produced by an endogenous enzyme of *A. oryzae* that reduced the ketone to an alcohol on the side chain. Modifications by endogenous enzymes were previously reported in *A. oryzae*. For example, undesired oxidations of biosynthetic intermediates were detected during heterologous biosynthesis of the polyketide solanapyrone in *A. oryzae*.^23^ Our results show that *A. oryzae* was able to express VdtA and produce the expected pyranone product 2.

### A Putative Biosynthetic Route to Xylindein

**Figure 7:**
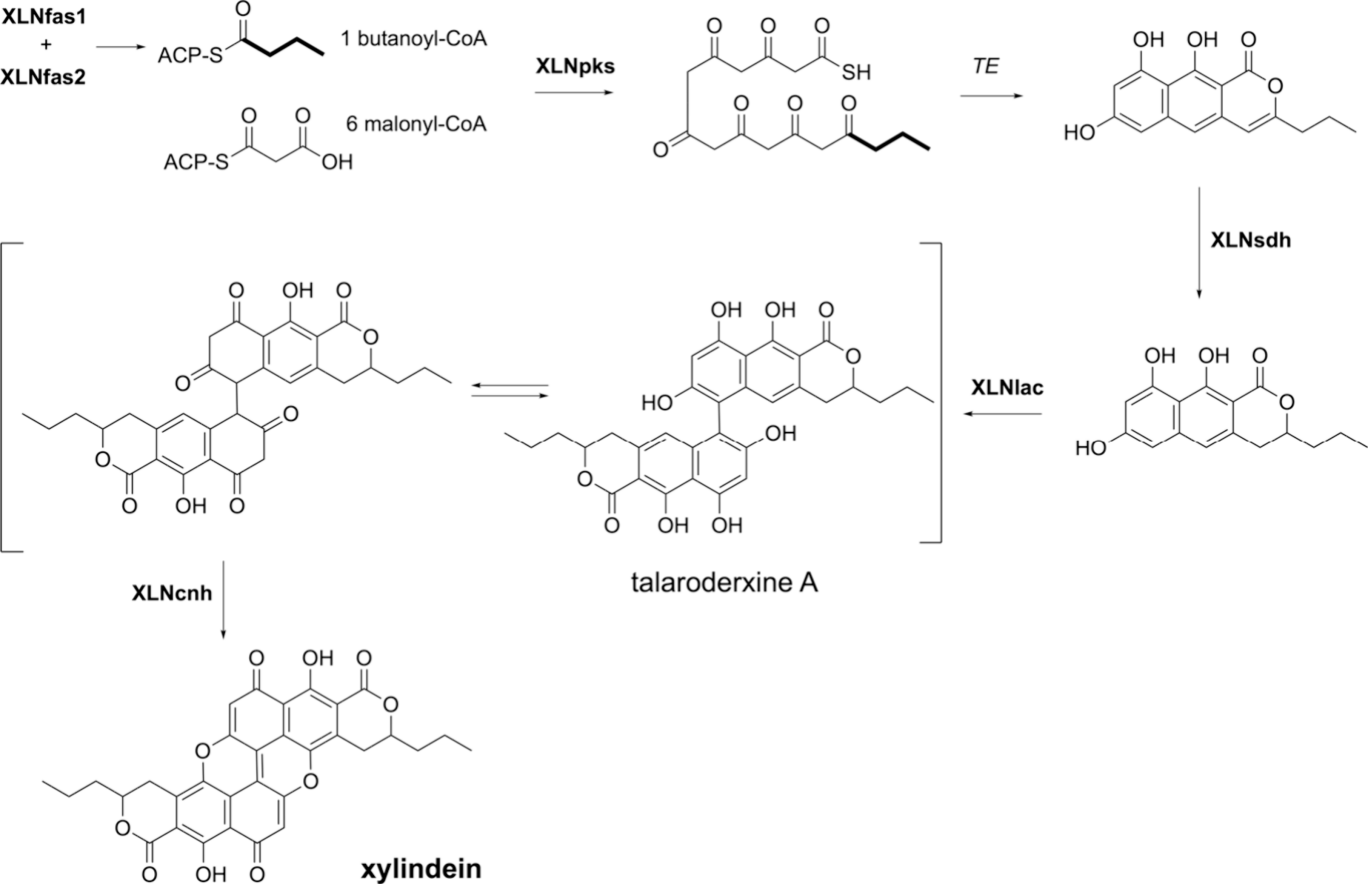
Proposed biosynthetic route of xylindein. XLNfas1 and XLNfas2 provide the precursor butanoyl-CoA to XLNpks. Taken along with 6 malonyl-CoA, XLNpks release the first intermediate, which is subsequently reduced by XLNsdh. XLNlac dimerizes the monomer with XLNcnh and produces the final product, xylindein.

Based on the genomic predictions and heterologous expression, we propose a biosynthetic pathway to yield xylindein. Several hypotheses could explain the absence of product when *XLNfas1*, *XLNfas2*, and *XLNpks* are co-expressed. First, the release mechanism of the polyketide from the nrPKS may require an extra enzyme as exemplified with group V nrPKSs that need a β-lactamase enzyme encoded within their BGCs.^24^ However, in the cercosporin biosynthetic pathway, it was shown that the release mechanism from the nrPKS Ctb1 is catalyzed by its TE domain that forms the pyrone.^25^ Thus, the observed pyrone ring in the structure of nor-toralactone, viriditoxin, and xylindein is expected to be the result of the releasing mechanism. A second hypothesis is a codon usage bias incompatible with *A. oryzae*. To evaluate this hypothesis, we calculated the codon adaptation index (CAI)^26^ between *vdtA*, *XLNpks*, *XLNfas1*, and *XLNfas2* genes with the host organism *A. oryzae*. The CAI scores range from 0.0 to 1.0, and genes with scores above 0.8 are considered to be suitable for heterologous expression.^27^ CAI scores are very similar for the three *XLN* and *vdtA* genes, and all are greater than 0.8 (Table S7), indicating that *C. aeruginascens* codon bias unlikely explains the absence of product. In the aflatoxin pathway, the starter unit hexanoyl-CoA is directly transferred from the FAS complex to the nrPKS.^22^ Thus, no other enzyme is expected to be required to produce the first intermediate from butanoyl-CoA and malonyl-CoA in the xylindein pathway. Based on these considerations, the first steps of the pathways are expected to be very similar to those of the aflatoxin and viriditoxin pathways (Figure 7). However, we cannot exclude that tailoring enzymes are required in these early steps in the biosynthetic pathway as found for sporothriolides, whose biosynthesis involves FASs (FasA and FasB) and a citrate synthase (SpoE).^28^ Co-expression of the three genes in *A. oryzae* did not yield any product, and the first stable intermediate was obtained only when the decarboxylase (spoK) and citrate dehydratase (spoL) were also co-expressed.^28^ There is no obvious candidate gene in the xylindein BGC that would encode a tailoring enzyme involved in the production of the expected first stable intermediate. Finally, the predicted starter unit butanoyl-CoA could be rapidly degraded by β-oxidation,^29^ preventing the XLNpks from using it, while the acetyl-CoA starter unit is easily available for VdtA and explains the successful expression in *A. oryzae*.

Similarities between xylindein and viriditoxin (Figure 1) suggest common tailoring of biosynthetic steps. XLNsdh likely exhibits a similar function to VdtF to reduce the pyrone ring,^17^ and we propose that XLNsdh will catalyze the first tailoring step in the xylindein pathway (Figure 7). It was demonstrated that VdtB catalyzes the dimerization step to yield viriditoxin.^17^ XLNlac is a multicopper oxidase homologous to VdtB and is thus expected to catalyze a similar phenol coupling in xylindein biosynthesis (Figure 7).^17^ The dimerization of xylindein is, however, unique because of additional C–O coupling (Figure 1). This feature suggests that the xylindein BGC should comprise a gene that is not found outside of the *Chlorociboria* genus (Figure 3). Although the predicted function is not compatible with the required oxidative chemistry, the only candidate is the putative carbonic anhydrase XLNcnh, which we propose to contribute to the final step of the xylindein biosynthesis (Figure 7). The exact enzymatic activity of XLNcnh remains to be determined with functional analyses.

In conclusion, through a combination of genome mining, phylogenetic dereplication, and expression analysis, this study identified the BGC as responsible for the production of xylindein, a valuable blue-green pigment derived from wood colonization by *Chlorociboria* species. Elucidation of the biosynthetic pathway requires further effort, especially to understand the early biosynthetic steps. Most importantly, the *A. oryzae* transformant expressing the viriditoxin nrPKS VdtA opens avenues to characterize xylindein tailoring enzymes, especially those responsible for the unique dimerization of xylindein.

## Experimental Section

### General Experimental Procedures

High-resolution mass spectrometry was performed on a Q-Tof Premier mass spectrometer (Waters) coupled to an Acquity UPLC system (Waters). Electron spray ionization (ESI) mass spectroscopy was measured in positive or negative mode depending on the compound. All solvents and chemicals used for HR-ESI-MS and chromatography were LC-MS grade, while the solvents for metabolite extraction were HPLC grade. Water was purified using a Milli-Q ultrapure water system.

### Fungal Strains and Growth Conditions

*C. aeruginascens* CBS 122017, *C. aeruginosa* CBS 139.28, and *C. aeruginosa* CBS 123.57 strains were grown and maintained on MEA plates at 21 °C. *P. variotii* CBS 101075 was grown and maintained on MEA plates at 30 °C. For genomic DNA extraction, strains were grown in 50 mL of MB (Table S8) liquid medium for 14 days at 21 °C under constant agitation at 200 rpm. For total RNA extraction, *C. aeruginascens* was grown in 50 mL of 5% OJ liquid medium (Table S8) at 21 °C under constant agitation at 200 rpm; mycelium was harvested at 6–8 days, 10–12 days, and 14–18 days when exhibiting different white, light blue, and dark blue pigmentation, respectively. *A. oryzae* NSAR1 was grown and maintained on MEA plates at 30 °C. *Saccharomyces cerevisiae* BMA 64 *Δura3* used for homologous recombination was grown and maintained on YPD plates at 30 °C.

### Nucleic Acid Extraction and RT-PCR

The mycelium of *Chlorociboria* species and *A. oryzae* transformants from liquid cultures was filtered through a paper filter, frozen in liquid nitrogen, and ground using a mortar and pestle. Genomic DNA of transformants was isolated using the DNeasy plant minikit (Qiagen, Hilden, Germany) according to the manufacturer’s recommendations. For genomic DNA for genome sequencing, 100 mg of the ground mycelium was mixed with 0.6 mL of warm cetyltrimethylammonium bromide (CTAB) buffer in a 1.5 mL microcentrifuge tube and incubated for 15 min at 65 °C. The resulting lysate was mixed with 0.6 mL of chloroform isoamyl alcohol (24:1) and violently mixed by vortex for 5 min. The sample was centrifuged at 12000*g* for 10 min at room temperature. The aqueous phase was transferred into a new microcentrifuge tube, incubated on ice for 10 min, and then mixed with 0.5 mL of cold isopropanol. The sample was centrifuged at 12000*g* for 15 min to obtain the pellet, followed by washing with 0.5 mL of cold ethanol and centrifugation for 10 min to remove the supernatant. The air-dried sample was eluted with 50 μL of water, mixed with 5 μL of RNase A (Thermo Fisher Scientific, Waltham, MA, USA), and incubated at 37 °C for 30 min. For total RNA extraction, 100 mg of the ground mycelium was mixed with xylindein and Invitrogen TRIzol reagent (Thermo Fisher Scientific) in a 1.5 mL microcentrifuge tube and incubated for 5 min at 25 °C. The resulting lysate was mixed with 0.2 mL of chloroform, gently mixed by hand, and incubated for 5 min. Samples were centrifuged at 12000*g* for 15 min at room temperature. The aqueous phase was transferred to a new microcentrifuge tube, mixed with 0.5 volume of 100% ethanol, and loaded into a column from the NucleoSpin RNA extraction kit (Macherey Nagel, Allentown, PA, USA). Downstream steps were performed according to the manufacturer’s protocol. Five micrograms of RNA was used to synthesize cDNA using oligo(dT) primers and GoScript reverse transcription (RT) mix (Promega, Madison, WI, USA) according to the manufacturer’s protocol. PCR was performed for *XLNpks*, *XLNfas1*, *XLNfas2*, and *vdtA* genes and the housekeeping control gene *H2B* using GoTaq DNA polymerase (Promega, Madison, WI, USA).

### Genome Sequencing, Assembly, and Gene Prediction

The genomic DNA was purified using 0.4 volume of AMPure XP Beads (Beckman Coulter, Brea, CA, USA). After the genomic DNA was mixed with AMPure XP beads, the suspension was incubated on a rotator mixer for 10 min at room temperature. The sample was spun down shortly and placed on a magnet for 5 min to pellet the beads. The supernatant was discarded, and the pellet was washed twice with 80% ethanol on the magnetic stand. Air-dried beads were resuspended in 35 μL of water and incubated at 37 °C for 10 min, gently flicking the sample every 2 min to encourage DNA elution. After the beads were spun down and pelleted on the magnet, the eluate was placed into a new tube. Purified genomic DNA samples were prepared for long-read sequencing with the Oxford Nanopore Technologies native barcoding kit SQK-NBD114.24 (ONT, Oxford, UK) according to the manufacturer’s protocol (version NBE_9769_v114_revI_15Sep2022, latest update 12/07/2023) using approximately 1 μg of DNA per sample. The final prepared libraries were sequenced on R10.4.1 flow cells (ONT) with the GridION sequencer (ONT). Basecalling of the reads was performed by Guppy with the high-accuracy basecalling model in Minknow. The first and last 50 bp of all “pass” reads were then chopped, using CHOPPER.^30^ The quality of the chopped reads was then checked using FastQC version 0.12.1,^31^ and Flye 2.9.2-b1786^32^ was used to assemble the chopped reads. The quality of the assembly was then checked using Quast version 5.2.0.^33^ Completeness of the assemblies was determined by a BUSCO v5 analysis using gVolante^34^ with the Leotiomycetes_odb10 data set. Genes were predicted using webAugustus^35^ with the *Botrytis cinerea* training set. Assemblies were visualized and telomeric regions predicted using Telovision (https://github.com/WesterdijkInstitute/TeloVision).

### Phylogenetic Trees and Comparative Genomics

Close homologues of *C. aeruginascens* XLNpks were retrieved from the Joint Genome Institute (JGI) MycoCosm repository^36^ using BLASTp against Pezizomycotina. Regions containing BGCs were retrieved using fungiSMASH 7.0 with default parameters.^21^ BGC comparison was performed using Clinker.^37^ Characterized nrPKSs were retrieved from the MIBiG database.^38^ Protein alignments were performed using MAFFT version 7.490^39^ (parameters–reorder) with nrPKS sequences. Poorly aligned regions were removed using trimaL version 1.4^40^ (build 2013–12–17; parameter -automated1). Maximum likelihood trees were built with IQ-TREE version 2.2.0-beta with model finder and ultrafast bootstrapping as well as an approximate likelihood-ratio test^41^ (parameters-mset LG -bb 1000 -alrt 1000 -T AUTO). The substitution model used for trees of nrPKS phylogenetic dereplication, XLNpks homologue phylogeny, and phylogeny of *Chlorociboria* nrPKSs is LG+F+R6, LG+R6, and LG+F+R6, respectively. The resulting trees were visualized using iTOL.^42^ All curated alignments and phylogenetic tree files are provided in the Supporting Information (Supplementary files S1–S6).

### Gene Amplification and Plasmid Digestion (*XLNpks, XLNfas1, XLNfas2, vdtA*)

*XLNpks* and *vdtA* were amplified from cDNA of *C. aeruginascens* and genomic DNA of *P. variotii*, respectively, using primers that harbor 30-bp sequences homologous to the *pEYA2* plasmid^43^ (Table S9). *XLNfas1* and *XLNfas2* were amplified from cDNA of *C. aeruginascens* using primers that contain 30-bp sequences homologous to the *pTYGSade* plasmid (behind promoters *Padh* and *Pgpd*; Table S9). All PCR fragments were amplified using Phusion high-fidelity DNA polymerase (Thermo Fisher Scientific) according to the manufacturer’s protocol. One microgram of the *pEYA2* plasmid or *pTYGSsde* plasmid was digested for 1 h with 10 U of *Not*I or *Asc*I (Promega, Madison, WI, USA), respectively, at 37 °C. Fragments of the expected size and the linearized plasmids were purified from a 0.8% agarose gel or directly from the PCR mix using a Geneclean II kit (MP Biomedicals, Santa Ana, CA, USA).

### Transformation-Associated Recombination in *Saccharomyces cerevisiae* (pEYA2::PKS, pTYGSade::FAS1::FAS2, pEYA2::vdtA)

A strain of *S. cerevisiae* BMA 64 with a *ura3*^*–*^ auxotrophic marker was used for transformation-associated recombination according to a protocol adapted from the one described previously.^44^*S. cerevisiae* was grown overnight at 30 °C in 3 mL of yeast YPD medium (Table S8). Two milliliters of culture containing 10^8^ cells was transferred into 50 mL of YPD medium and incubated at 30 °C under agitation at 200 rpm for about 5 h until reaching an optical density at 600 nm (OD_600_) of 1 to 1.5. Yeast cells were centrifuged 5 min at 2500 rpm at 4 °C, and the supernatant was discarded. Fifty microliters of cells was mixed with 250 μL of DTT (100 mM) and incubated for 10 min at room temperature. The mixture was centrifuged for 15 s, and the supernatant was discarded. The pellet was gently mixed with 500 μL of PLTE buffer (800 μL of 50% PEG, 100 μL of 1 M LiAc, 20 μL of 50 mM XXX, 10 μL of 1 M Tris HCl pH 7.5, 70 μL of H_2_O), 4 μL of each DNA fragment and digested plasmid, and 50 μL of boiled salmon sperm DNA and incubated for 1 h at 30 °C with inverting the tube once every 20 min. After 1 h of incubation, cells were heat shocked at 45 °C for 15 min. The pellet was collected by centrifuging for 15 s, and the supernatant was discarded. Cells were gently resuspended in 1 mL of sterile water and centrifuged for 15 s to remove the water. The pellet was resuspended in 1 mL of YPD liquid medium and incubated at 30 °C for 30 min without shaking. Cells were collected after 15 s of centrifugation. The pellet was reconstituted in 200 μL of sterile water and plated on SDM plates (Table S8). Plates were incubated for 3 to 7 days at 30 °C. Yeast transformants were transferred to a new SDM plate and grown overnight. Single colonies were transferred into a microcentrifuge tube in 30 μL of 25 mM NaOH and boiled for 10 min at 100 °C. Next, 1 mL was used for PCR screening with GoTaq DNA polymerase (Promega, Madison, WI, USA) and the corresponding cloning primers (Table S9). Positive transformants were grown overnight in liquid SDM to isolate the plasmid using the Zymoprep yeast plasmid miniprep kit (Zymo Research, Irvine, CA, USA). The obtained plasmids were subsequently introduced into electrocompetent *Escherichia coli* DH5a cells (for *pEYA2::PKS* and *pEYA2::vdtA*; Thermo Fisher Scientific) and 2T1 cells (for *pTYGSade::FAS1::FASs2;* Thermo Fisher Scientific) using an electroporation method according to the manufacturer’s protocol (electroporator conditions: 2.0 kV, 200 Ω, 25 μF). PCR screening was performed by transferring individual colonies into the PCR mixture with the GoTaq DNA polymerase (Promega). The plasmid was isolated from confirmed positive clones using the Zyppy plasmid miniprep kit (Zymo Research), and the plasmid was validated by sequencing (Macrogen, Seoul, South Korea) with sequencing primers (Table S9). Plasmid *pTYGSade::FAS1::FASs2* was subsequently isolated from a sequence confirmed clone by the HiPure plasmid midiprep kit (Thermo Fisher Scientific).

### Construction of the Expression Vector (*pTYGSarg::PKS*, *pTYGSade::vdtA*)

Seventy nanograms of the *pEYA2::PKS* or *pEYA2::vdtA* entry vector and 100 ng of the *pTYGSarg* or *pTYGSade* destination vector^38^ were mixed with 1 μL of the Gateway LR Clonase II enzyme (Thermo Fisher Scientific) in a 5 μL final volume, and the reaction mixture was incubated at 25 °C for 2 h. The total reaction mixture was introduced into chemically competent *E. coli* DH5a cells (Thermo Fisher Scientific) using a heat shock protocol. The *pTYGSarg::PKS* and *pTYGSade::vdtA* expression vectors were isolated from positive colonies using the Zyppy plasmid miniprep kit (Zymo Research) and the HiPure plasmid midiprep kit (Thermo Fisher Scientific) for *A. oryzae* transformation.

### Transformation of *A. oryzae* NSAR1

Spores from *A. oryzae* NSAR1 were harvested from MEA plates in 5 mL of sterile water, and 1 mL of this spore suspension was inoculated into 50 mL of MB liquid medium and grown overnight at 28 °C with shaking at 200 rpm. Germinating spores were collected by centrifugation at room temperature for 10 min at 3500 rpm and resuspended in 25 mL of 0.8 M NaCl. After spinning down for 10 min at 3500 rpm at room temperature, germinated spores were resuspended in 10 mL of a freshly made filter-sterilized protoplasting solution (200 mg of Trichoderma lysing enzyme (Thermo Fisher Scientific) and 50 mg of Driselase (Thermo Fisher Scientific) in 0.8 M NaCl) and incubated at 30 °C for 2 to 2.5 h with shaking at 100 rpm. Protoplasts were filtered through sterile Miracloth and then centrifuged for 5 min at 3000 rpm at 4 °C. Protoplasts were resuspended in 200 μL of solution 1 (0.8 M NaCl, 10 mM CaCl_2_, and 50 mM Tris-HCl pH 7.5) and aliquoted to 100 μL in 2 mL microcentrifuge tubes. Ten micrograms of the *pTYGSarg::PKS*, *pTYGSade::FAS1::FAS2*, and *pTYGSade::vdtA* expression plasmids or *pTYGSarg* and *pTYGSade* empty vectors were added to protoplasts, and the mixture was incubated on ice for 2 min. One milliliter of solution 2 (60% (wt/vol) polyethylene glycol 3350, 0.8 M NaCl, 10 mM CaCl_2_, and 50 mM TrisHCl pH 7.5) was added, and the tubes were gently inverted before incubation at room temperature for 20 min. Protoplasts were then mixed with 25 mL of cooled Top CZD agar A, B, or C (Table S8) and immediately plated onto the corresponding Bottom CZD agar plates (Table S8). Transformation plates were incubated at 30 °C for 3 to 10 days. Transformants were transferred onto new selection CZD agar plates individually and transferred onto DPY agar plates (Table S8) for induction.

### Secondary-Metabolite Extraction, HPLC, and HRMS Analyses

The 4-day-old *A. oryzae* transformant on DPY agar plates was cut, collected into the 50 mL tubes, and isolated with ethyl acetate (VWR Chemicals, Radnor, PA, USA) for screening the metabolic profile. After shaking on an orbital shaker for at least 1 h, the organic phase was transferred to a new 50 mL tube and evaporated under nitrogen flow. For the 50 mL DPY broth culture, secondary metabolites from 4-day-old transformant liquid culture filtrates were isolated with a 1:1 volume of ethyl acetate, followed by the same shaking and evaporating steps. The resulting solid was dissolved in acetonitrile. Organic extracts were analyzed with a Shimadzu LC-2030 3D Prominence-i PDA system coupled to a Shimadzu LCMS-2020 mass spectrometer and equipped with a Shimadzu Shim-pack GIST C_18_-HP reversed-phase column (3 mm, 4.6 mm × 100 mm). The following method was used: a linear gradient of buffer B (5% to 95%) for 10 min, 2 min of 95% buffer B, gradient of buffer B (95% to 5%) for 1 min, and then 5% buffer B for 5 min. Water with 0.1% trifluoroacetic acid (TFA) for high-performance liquid chromatography (HPLC) or 0.05% formic acid for mass spectrometry (MS)-coupled analyses was used as buffer A, and acetonitrile (LCMS grade) with 0.1% TFA for HPLC or 0.05% formic acid for MS-coupled analyses was used as buffer B. The flow rate was 1 mL/min or 0.5 mL/min for UV-HPLC or MS-coupled analyses, respectively. The equipment was controlled and results were analyzed using Shimadzu LabSolutions LCMS software. HRMS was performed on a Q-Tof Premier mass spectrometer (Waters) coupled to an Acquity UPLC system (Waters). ESI mass spectroscopy was measured in positive or negative mode depending on the compound. The chemical formulas of product 1 and product 2 were determined using the measured exact mass on the ChemCalc server.^45^ MarvinSketch 24.3.0 was used to draw chemical structures and generate preferred IUPAC names (https://www.chemaxon.com).

### RNA Sequencing

Library preparation was performed using a custom barcoding protocol as previously described,^46^ before continuing with the protocol of the Oxford Nanopore direct RNA sequencing kit (SQK-RNA002). Concentration of each individually barcoded sample was determined using a Qubit RNA high-sensitivity kit (Invitrogen, Q32852). Samples were then pooled, and the final concentration was determined using Qubit (Invitrogen, Q32851). RNA of *S. cerevisiae* Enolase II was used as a spike-in. Between 50 and 300 ng of the libraries was sequenced on the Oxford Nanopore MinION Mk1C with R9 flow cells, and the sequencing runs were monitored using the accompanying MinKNOW 23.07.12 software.^47^ Base-calling was performed with Guppy version 7.1.4. Demultiplexing was performed with the ‘resnet20-final.h5’ barcode prediction model according to the custom barcoding protocol with the CPU method described in Deeplexicon.^46^ The spike-in reads were aligned using Minimap2 (2.24).^48^ The spike-in and sample reads were separated using Samtools (1.18) view (-F4 -hS and -f4 -hS).^49^ The latter was converted to a fastq format using the Samtools bam2fq command. The fastq files were combined to generate a single file according to experimental conditions and biological and technical replications. The total reads were then mapped to the reference genome of *C. aeruginascens* CBS 122017 using Minimap2.^48^ Expression was quantified using Featurecounts (Subread 2.0.6)^50^ with *C. aeruginascens* gene prediction.

### Codon Adaptation Index Calculation

To calculate the CAI, the codon usage table for *A. oryzae* was acquired from the Codon Usage Database (https://www.kazusa.or.jp/codon/). This table was then fed into two calculators, OPTIMIZER and E-CAI from CAIcal.^27^ The CAI of the genes was evaluated by submitting exonic sequences only.

## Data Availability

Published sequence and assembly of *C. aeruginasens* DSM 107184 is on NCBI with the accession of NCSK00000000.2. Other sequencing data and assemblies are available on NCBI under the umbrella PRJNA1062589 bioproject. This Whole Genome Shotgun project has been deposited at DDBJ/ENA/GenBank under the accessions JBAWJB000000000, JBAWJC000000000, and JBAWJD000000000. The version described in this paper is version JBAWJB010000000, JBAWJC010000000, and JBAWJD010000000. The xylindein BGC locus is available at NCBI under the GenBank accession number PP549521.1.

## Supporting Information Available

Additional experimental details, methods, and analysis, including supplementary figures and tables (PDF)
Amino acid sequences, alignments, and tree file mentioned in the main text; RNAseq data (ZIP)
