# Supplementary material for: Identification of a Biosynthetic Gene Cluster for the Production of the Blue-Green Pigment Xylindein by the Fungus Chlorociboria aeruginascens
Source: J Nat Prod. 2025 Jan 23;88(2):233–44. doi: 10.1021/acs.jnatprod.4c00350 (PMC11877519; doi:10.1021/acs.jnatprod.4c00350)
Supplement: Supplementary file 1 — np4c00350_si_001.pdf [file np4c00350_si_001.pdf]

Table of Contents

Supplementary files

Supplementary file S1. nrPKS sequences used in the phylogenetic dereplication.

Supplementary file S2. Trimmed alignment of nrPKSs used in the phylogenetic dereplication.

Supplementary file S3. Tree file of the phylogenetic dereplication.

Supplementary file S4. nrPKS sequences used to build the phylogeny of *Chlorociboria* nrPKSs.

Supplementary file S5. Trimmed alignment of *Chlorociboria* nrPKSs.

Supplementary file S6. Tree file of *Chlorociboria* nrPKSs.

Supplementary file S7. RNAseq quantification of gene expression in *Chlorociboria aeruginascens*.

Supplementary figures

Figure S1. Genome assemblies visualized by TeloVision. 2

Figure S2. Phylogenetic tree of nrPKSs from *C. aeruginascens, C. aeruginosa,* and characterized nrPKSs. 3

Figure S3. Characterization of *AoADE::vdtA* transformants. 4

Figure S4. HRMS analysis of product 1 and product 2. 5

Figure S5. ^{1}H NMR analysis of product 1 6

Supplementary tables

Table S1. *Chlorociboria aeruginascens* and *Chlorociboria aeruginosa* genome assembly statistics. 7

Table S2. Prediction of core biosynthetic proteins (CBPs). 8

Table S3. Conserved domain organization of predicted non-reducing polyketides synthases. 9

Table S4. BlastP search results for proteins encoded at the *XLNpks* locus. 10

Table S5. Level of conservation between conserved enzymes encoded at the *XLNpks* locus in different species. 12

Table S6. Number of transformants obtained in this study. 13

Table S7. Codon adaptation index (CAI) scores of *VdtA, XLNpks, XLNfas1, XLNfas2* genes in *A. oryzae* 14

Table S8. Media used in this study. 15

Table S9. Primers used in this study. 17

Supplementary figures

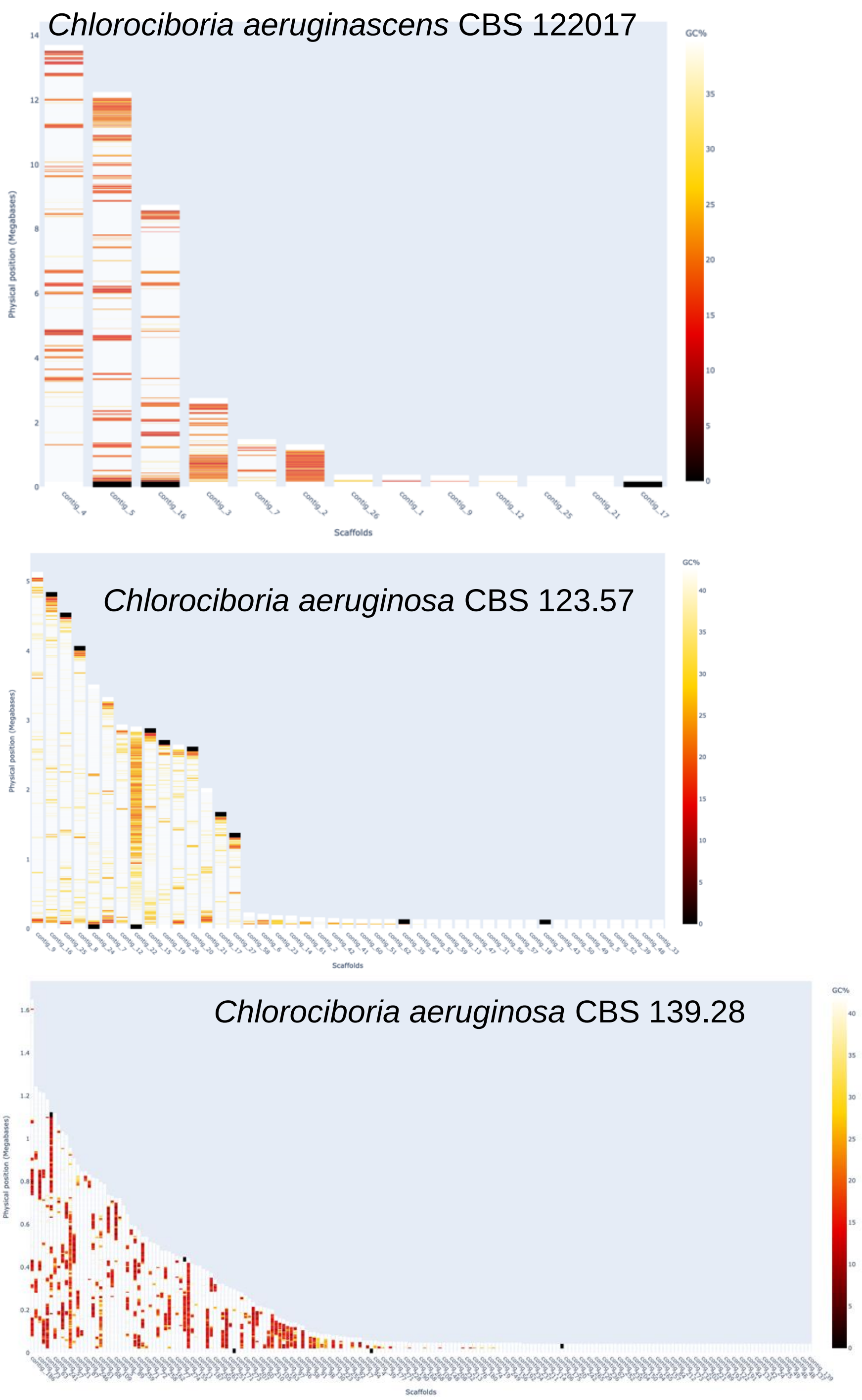

Figure S1. Genome assemblies visualized by TeloVision. Each scaffold is drawn to scale and the GC content is indicated with the color scale. Detected telomeric regions are visualized as black boxes at the ends of scaffolds, while the absence of telomeres is indicated with a white box at the end of scaffolds. The consensus telomeric repeat detected in all three strains is CTAACC. However, three telomeric regions in *C. aeruginosa* CBS 139.28 consist of different low complexity repeats (AGAGAGAGA, TCTCTCTC or CTCGTC) and are unlikely true telomeres. *Chlorociboria* species likely comprise between four and six chromosomes.

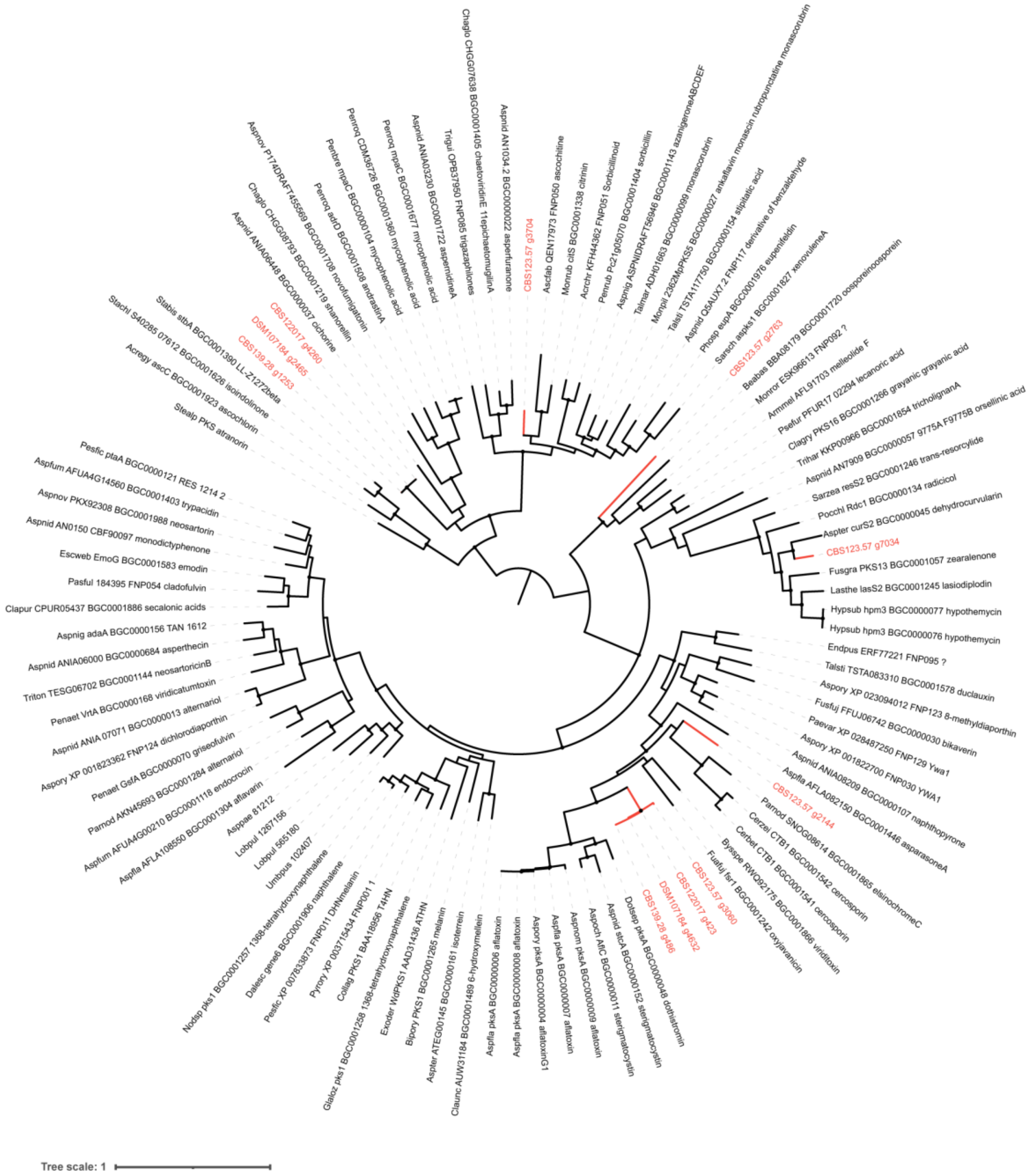

Figure S2. Phylogenetic tree of non-reducing polyketide synthases (nrPKSs) from *C. aeruginascens*, *C. aeruginosa*, and characterized nrPKSs. All nrPKSs from *C. aeruginascens* and *C. aeruginosa* were highlighted in red. Ultrafast bootstrap values over 95 are indicated with black dots at the nodes. The tree is midpoint rooted. g3060 from strain CBS123.57, g486 from CBS139.28, g4632 from DSM 107184 and g423 from CBS 122017 are the unique shared nrPKS candidates for xylindein production.

A

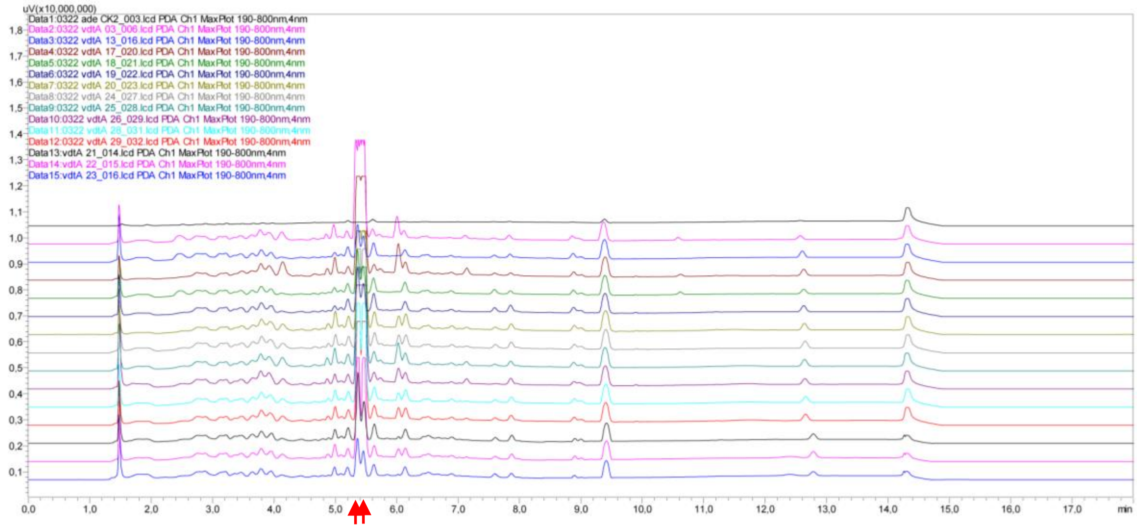

B

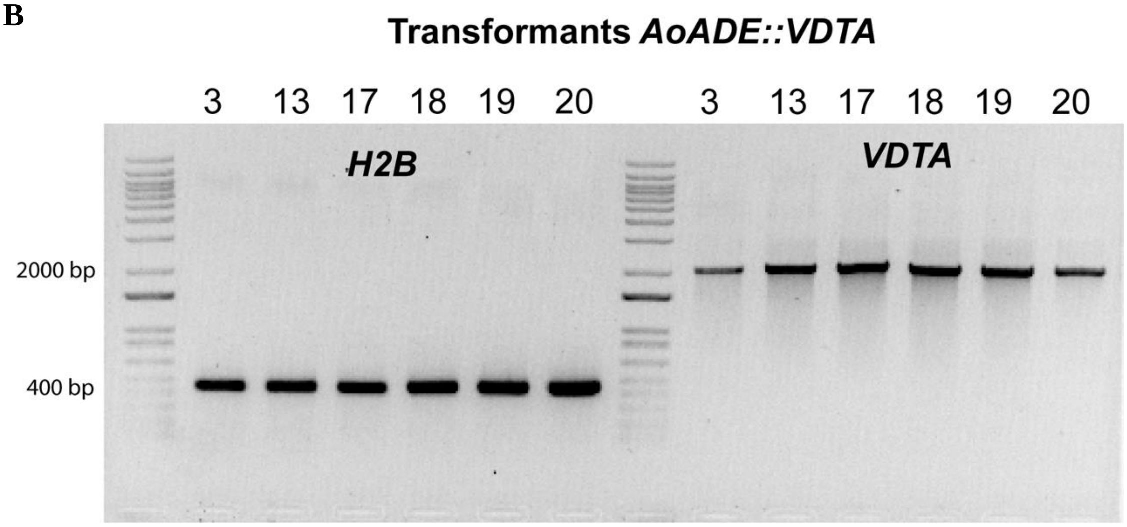

Figure S3. Characteirzation of *AoADE::vdtA* transformants. (A) First UV-HPLC trace is the control transformed with empty vector and other traces are transformants expressing the *vdtA* gene. Red arrows indicate the two major products that are not present in the control. **(B)** Gene expression of the selected transformants as determined by RT-PCR. The *H2B* housekeeping gene was used as an expression control.

Product 1

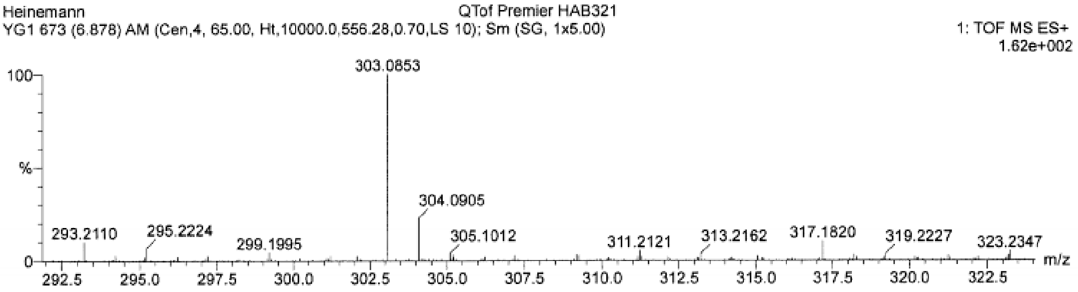

#	MF	Monoisotopic mass	PPM	mDa	unsaturation
Filter data	Filter data	min - max	min - max	min - max	min - max
1	$C_{16}H_{15}(N_0)O_6$	303.0869	-5.16	-1.56	9.5
2	$C_{23}H_{11}(N_0)O$	303.0810	14.22	4.31	18.5
3	$C_9H_{19}(N_0)O_{11}$	303.0927	-24.54	-7.44	0.5
4	$C_{12}H_{15}(N_0)O_9$	303.0716	45.18	13.69	5.5
5	$C_{20}H_{15}(N_0)O_3$	303.1021	-55.49	-16.82	13.5
6	$C_{19}H_{11}(N_0)O_4$	303.0657	64.56	19.57	14.5

Product 2

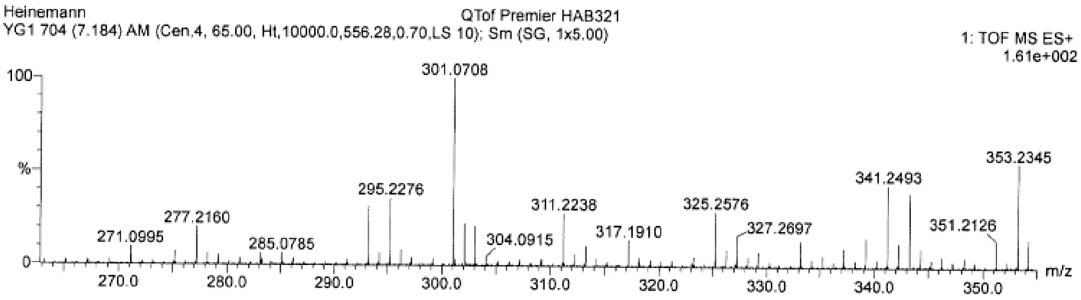

#	MF	Monoisotopic mass	PPM	mDa	unsaturation
Filter data	Filter data	min - max	min - max	min - max	min - max
1	$C_{16}H_{13}(N_0)O_6$	301.0712	-1.37	-0.41	10.5
2	$C_{23}H_9(N_0)O$	301.0653	18.14	5.46	19.5
3	$C_9H_{17}(N_0)O_{11}$	301.0771	-20.88	-6.29	1.5
4	$C_{12}H_{13}(N_0)O_9$	301.0560	49.30	14.84	6.5
5	$C_{20}H_{13}(N_0)O_3$	301.0865	-52.04	-15.67	14.5
6	$C_{19}H_9(N_0)O_4$	301.0501	68.81	20.72	15.5

Figure S4. HRMS analysis of product 1 and product 2. The chemical formula were determined using the measured exact mass on the ChemCalc server.

A ChemNMR ^{1}H Estimation

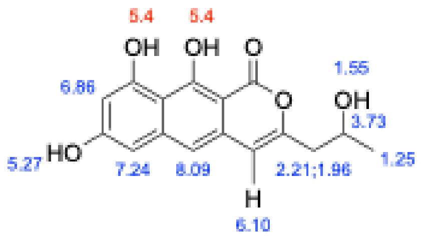

Product 1:

7,9,10-trihydroxy-3-(2-hydroxypropyl)-1H-benzo[g]isochromen-1-one

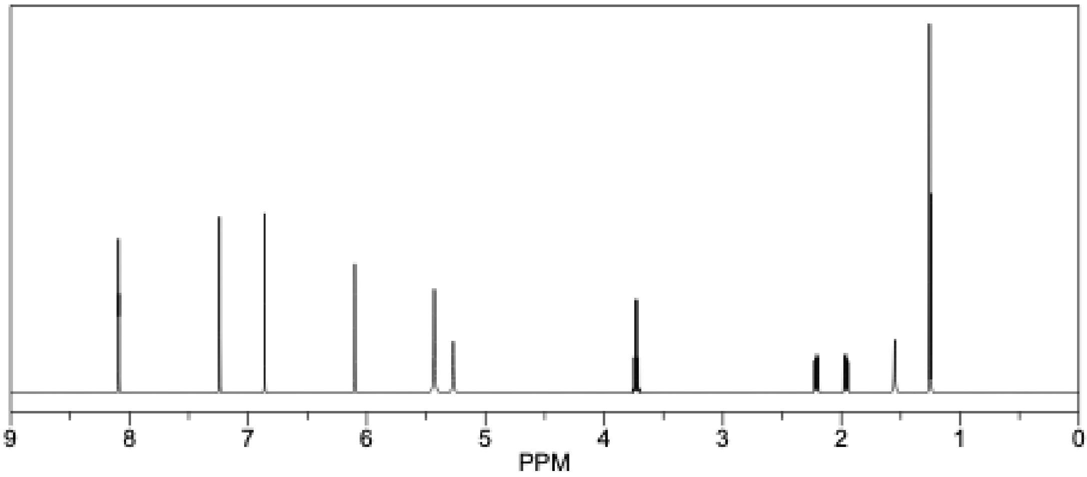

B

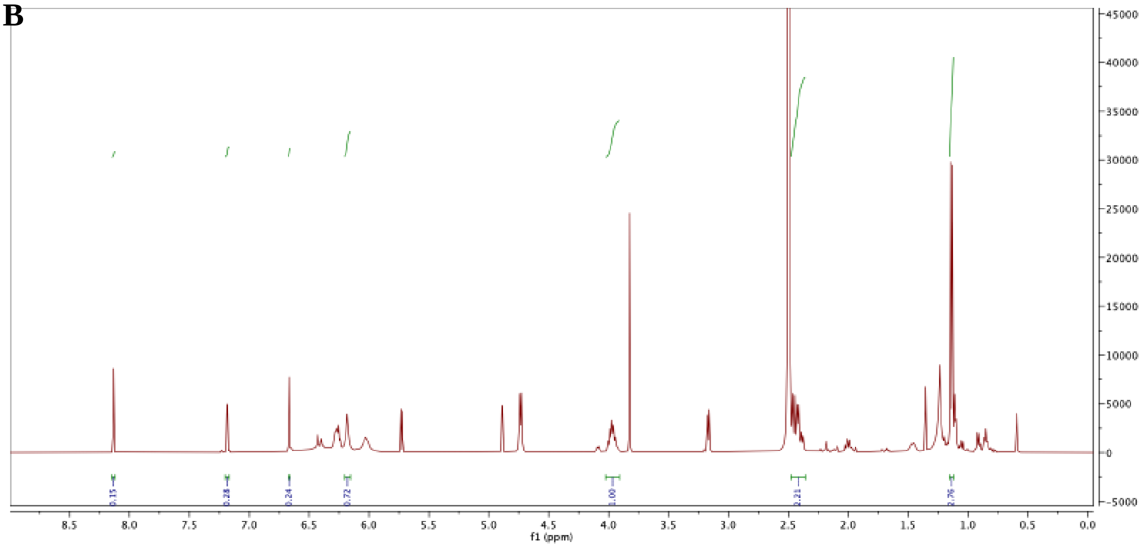

^{1}H NMR (400 MHz, DMSO) δ 8.13 (s, 1H), 7.18 (d, J = 1.2 Hz, 1H), 6.67 (s, 1H), 6.18 (s, 1H), 3.97 (dt, J = 12.1, 6.2 Hz, 1H), 2.42 (td, J = 14.7, 6.4 Hz, 2H), 1.14 (d, J = 6.1 Hz, 3H).

Figure S5. ^{1}H NMR analysis of product 1. (A) ^{1}H NMR (CDCl3 600 MHz) trace of product 1 as predicted by ChemDraw. **(B)** ^{1}H NMR trace obtained for product 1.

Supplementary tables

Table S1. *Chlorociboria aeruginascens* and *Chlorociboria aeruginosa* genome assembly statistics. The assembly of *C. aeruginascens* DSM 107184 was previously published.

**Strain**	***C. aeruginascens* DSM 107184**	***C. aeruginascens* CBS 122017**	***C. aeruginosa* CBS 123.57**	***C. aeruginosa* CBS 139.28**
Technology	Illumina	Nanopore	Nanopore	Nanopore
Assembly size	33.1 Mb	38.3 Mb	45.9 Mb	38.9 Mb
Coverage	39.0X	49.6X	52.3X	17.8X
Contigs	588	13	44	205
N50	110,634	11,901,332	3,206,439	748,623
L50	99	2	6	20
L90	NA	4	13	55
GC%	43.1	39.62	42.15	38.97
BUSCO summary[a]	97.7%	97.3 %	97.2 %	95.5 %
Coding genes[b]	8,648	9,457	12,210	9,630

[a] BUSCO analysis was performed with gVolante

[b] Genes were predicted using webAugustus

Table S2. Prediction of core biosynthetic proteins (CBPs). The assembly of *C. aeruginascens* DSM 107184 was previously published. Regions with predicted biosynthetic gene clusters (BGCs) were predicted with antiSMASH 7.0, and reclassified using BGCtoolkit (https://github.com/WesterdijkInstitute/BGClib). PKS: polyketide synthase; NRPS: non-ribosomal peptide synthetase; TC: terpene cyclase; CoA: coenzyme A.

	C. aeruginascens **DSM 107184**	***C. aeruginascens*** **CBS 122017**	***C. aeruginosa*** **CBS 123.57**	***C. aeruginosa*** **CBS 139.28**
Total BGCs	46	48	57	44
Total CBPs	56	59	69	57
non-reducing PKS	2	2	5	2
reducing PKS	12	12	14	12
PKS-NRPS	1	1	1	1
NRPS	5	5	6	6
CoA ligases (NRPS-like)	12	14	14	12
TC	5	10	9	11
other	14	14	20	13

Table S3. Conserved domain organization of predicted non-reducing polyketides synthases. SAT: Starter Acyl-carrier protein Transacylase; KS: Keto-Synthase: AT: Acyl-Transferase; PT: Product Template; T: Thiolation; ACP: Acyl-Carrier Protein; TE: ThioEsterase; HTH: Helix-turn-helix; CMeT: C-methyl transferase; NAD: Nicotinamide Adenine Dinucleotide; FAD: Flavin Adenine Dinucleotide.

Strain	antiSMASH region	Gene ID	Domain architecture
C. aeruginascens CBS 122017	fungismash.contig_4.region007	g4260	SAT | KS | KS_C | AT | DH | T/ACP | T/ACP | Abhydrolase_3 | Peptidase_S9
	fungismash.contig_16.region003	g423	SAT | KS | KS_C | AT | PT | T/ACP | T/ACP | TE
C. aeruginosa CBS 139.28	fungismash.contig_101.region001	g486	SAT | KS | KS_C | AT | PT | T/ACP | T/ACP | TE
	fungismash.contig_11.region001	g1253	SAT | KS | KS_C | AT | DH | T/ACP | T/ACP | Abhydrolase_3 | Peptidase_S9
C. aeruginosa CBS 123.57	fungismash.contig_16.region004	g2144	SAT | KS | KS_C | AT | PT | T/ACP | TE
	fungismash.contig_16.region009	g2763	SAT | KS | KS_C | AT | PT | T/ACP | TE
	fungismash.contig_17.region002	g3060	adh_short | SAT | KS | KS_C | AT | PT | T/ACP | T/ACP | TE
	fungismash.contig_19.region004	g3704	SAT | KS | KS_C | AT | T/ACP | T/ACP | HTH_51 | CMeT | NAD_binding_4 | FAD_binding_3
	fungismash.contig_25.region003	g7034	SAT | KS | KS_C | AT | PT | T/ACP | TE

Table S4. BlastP search results for proteins encoded at the *XLNpks* locus. Hits in green correspond to conserved homologous loci. Hits in red are distant homologues.

**Protein query**	**Best BlastP hit in *Polyphilus sieberi* (e-value|%identity|%coverage)**	**Best BlastP hit in *Hysterium pulicare* (e-value|%identity|%coverage)**	**Best BlastP hit in *Stagonospora sp.* (e-value|%identity|%coverage)**	**Best BlastP hit in *Flavomyces fulophazii* (e-value|%identity|%coverage)**	**Best BlastP hit in *Periconia macrospinosa* (e-value|%identity|%coverage)**
g421	788440 (0.0e0|79|92) scaffold 15	115568 (1.80e-78|58|99) scaffold 239	122177 (3.51e-78|57|96) scaffold 5	658531 (2.52e-79|58|99) scaffold 104	619275 (1.42e-77|57|99) scaffold 290
g422	726361 (3.76e-180|61|93) scaffold 15	120174 (2.01e-34|42|59) scaffold 456	290194 (1.16e-35|43|59) scaffold 2	94634 (8.47e-38|40|59) scaffold 15	667290 (2.68e-36|37|64) scaffold 8
g423 *pks*	872537 (0.0e0|64|100) scaffold 16	121385 (0.0e0|70|100) scaffold 572	234140 (0.0e0|67|97) scaffold 21	739674 (0.0e0|65|100) scaffold 47	698930 (0.0e0|65|100) scaffold 36
g424 *sdh*	789957 (3.62e-108|71|100) scaffold 16	121383 (6.34e-163|80|100) scaffold 572	298559 (1.10e-156|76|100) scaffold 21	710374 (2.33e-159|77|100) scaffold 47	521457 (3.54e-157|76|100) scaffold 36
g425 *lac*	658105 (1.57e-109|64|89) scaffold 16	121384 (1.40e-12-|67|93) scaffold 572	321074 (8.01e-101|63|87) scaffold 21	616058 (7.19e-101|64|86) scaffold 47	521543 (6.71e-101|64|97) scaffold 36
g426 *cnh*	303359 (4.20e-26|42|82) scaffold 25	121984 (1.32e-33|45|26) scaffold 583	333352 (1.36e-6|40|23) scaffold 3	No hit	635458 (7.17e-10|42|27) scaffold 396
g427 *tf3*	638301 (4.32e-73|60|90) scaffold 4	121983 (1.51e-16|49|49) scaffold 583	38731 (1.21e-7|25|22) scaffold 13	730149 (3.22e-19|37|23) scaffold 16	695211 (5.01e-12|36|28) scaffold 272

g428<br>*met*	748822 (3.04e-31|31|60)<br>scaffold 16	111424 (6.08e-16|32|20)<br>scaffold 106	292291 (3.09e-15|29|50)<br>scaffold 5	631741 (2.57e-22|41|38)<br>scaffold 7	670136 (9.22e-23|43|37)<br>scaffold 37
g429<br>*tf2*	166291 (3.25e-25|60|49)<br>scaffold 16	121387 (4.97e-23|61|58)<br>scaffold 572	382208 (9.93e-20|54|58)<br>scaffold 21	299616 (7.17e-21|60|45)<br>scaffold 47	612484 (1.48e-38|57|41)<br>scaffold 36
g430<br>*tf1*	618621 (4.77e-98|59|87)<br>scaffold 16	121388 (4.88e-54|56|90)<br>scaffold 572	86029 (3.27e-79|57|67)<br>scaffold 21	562513 (8.81e-81|54|83)<br>scaffold 47	268080 (5.56e-80|56|78)<br>scaffold 36
g431<br>*fas2*	891520 (0.0e0|60|91)<br>scaffold 16	121381 (0.0e0|62|81)<br>scaffold 572	286687 (9.42e-165|59|82)<br>scaffold 21	299705 (2.73e-174|60|83)<br>scaffold 47	268045 (6.05e-173|59|84)<br>scaffold 36
g432<br>*fas1*	693732 (0.0e0|56|86)<br>scaffold 16	121382 (0.0e0|56|89)<br>scaffold 572	353560 (0.0e0|53|86)<br>scaffold 21	689350 (0.0e0|54|89)<br>scaffold 47	558682 (0.0e0|55|94)<br>scaffold 36
g433	739970 (1.20e-102|82|91)<br>scaffold 4	112596 (5.09e-51|68|65)<br>scaffold 14	288481 (9.52e-75|70|64)<br>scaffold 1	709703 (9.23e-76|67|59)<br>scaffold 37	569438 (1.09e-74|71|60)<br>scaffold 163
g434	765476 (1.32e-108|73|85)<br>scaffold 4	112597 (1.61e-17|60|60)<br>scaffold 14	311761 (9.55e-15|54|61)<br>scaffold 1	737218 (1.75e-15|56|57)<br>scaffold 37	117331 (8.62e-16|56|57)<br>scaffold 163

Table S5. Level of conservation between conserved enzymes encoded at the *XLNpks* locus in different species. Amino acid identity percentage compared to *C. aeruginascens* was determined using Clinker.

C. aeruginascens	*C. aeruginosa* CBS 139.28	*C. aeruginosa* CBS 123.57	*P. macrospinosa*	*F. fulophazii*	*Stagonospora sp.*	*H. pulicare*	*P. sieberi*	*A. flavus*	*P. variotii*	*F. fujikuroi*	*C. beticola*
g432 *fas1*	1.00	0.85	0.48	0.49	0.48	0.51	0.49	0.38	< 0.3	< 0.3	< 0.3
g431 *fas2*	0.99	0.89	0.51	0.5	0.51	0.54	0.56	0.44	< 0.3	< 0.3	< 0.3
g430 *tf1*	0.96	0.79	0.49	0.5	0.46	< 0.3	0.47	< 0.3	< 0.3	< 0.3	< 0.3
g429 *tf2*	1.00	0.61	0.42	0.4	0.4	0.43	0.39	< 0.3	< 0.3	< 0.3	< 0.3
g428 *met*	1.00	0.88	< 0.3	< 0.3	< 0.3	< 0.3	< 0.3	< 0.3	0.4	< 0.3	< 0.3
g426 cnh	0.98	0.70	< 0.3	< 0.3	< 0.3	< 0.3	< 0.3	< 0.3	< 0.3	< 0.3	< 0.3
g425 *lac*	0.97	0.82	0.54	0.57	0.57	0.62	0.57	< 0.3	0.47	< 0.3	0.33
g424 *sdh*	0.99	0.98	0.77	0.77	0.76	0.8	0.71	< 0.3	0.38	< 0.3	< 0.3
g423 *pks*	0.98	0.96	0.62	0.61	0.61	0.66	0.59	0.46	0.45	0.35	0.41

Table S6. Number of transformants obtained in this study.

Name	Total number of obtained transformants	Number of transformants producing additional compounds / Number of transformants screened by HPLC	Number of transformants expressing introduced genes / Number of transformants tested by RT-PCR
AoARG::PKS	44	0/44	7/11
AoADE::FAS1::FAS2	17	0/17	6/10
AoADEARG::FAS1::FAS2::PKS	49	0/49	4/15
AoARGADE::PKS::FAS1::FAS2	35	0/35	4/12
AoADE::vdtA	55	14/30	4/4

Table S7. Codon adaptation index (CAI) scores of *VdtA, XLNpks, XLNfas1 and XLNfas2* genes in *Aspergillus oryzae*. CAI scores were determined using Optimizer and E-CAI calculators from CAI-cal.

Gene	Original organism	OPTIMIZER	E-CAI
vdtA	*P. variotii*	0.838	0.855
XLNpks(g423)	*C. aeruginascens*	0.843	0.835
XLNfas1(g432)	*C. aeruginascens*	0.814	0.846
XLNfas2(g431)	*C. aeruginascens*	0.809	0.822

Table S8. Media used in this study.

Media	Recipe		Brand
MEA	50 g/L Malt extract agar, pH 5.4 ± 0.2.		oxoid cm59
MB	30 g/L malt extract, pH 5.4 ± 0.2.		oxoid L39
5% OJ broth	5 ml/L orange juice		
5% OJ agar	5 ml/L orange juice, 15 g/L agar.		
Top CZD agar (A, B, D)	Base: 35 g/L Czapek-Dox, 1 g/L ammonium sulfate, 182.1g/L1M Sorbitol, 8 g/L selective agar.	A: 0.5 g/L adenine, 1.5 g/L methionine	Sigma-Aldrich, St. Louis, MO.
		B: 1 g/L arginine, 1.5 g/L methionine	
		D: 1.5 g/L methionine	
Bottom CZD agar (A, B, D)	Base: 35 g/L Czapek-Dox, 1 g/L ammonium sulfate, 182.1g/L1M Sorbitol, 15 g/L selective agar.	A: 0.5 g/L adenine, 1.5 g/L methionine	Sigma-Aldrich, St. Louis, MO.
		B: 1 g/L arginine, 1.5 g/L methionine	
		D: 1.5 g/L methionine	
Re-selection CZD agar (A, B, D)	Base: 35 g/L Czapek Dox, 1 g/L ammonium sulfate, 15 g/L selective agar.	A: 0.5 g/L adenine, 1.5 g/L methionine	Sigma Aldrich, St. Louis, MO.
		B: 1 g/L arginine, 1.5 g/L methionine	
		D: 1.5 g/L methionine	
DPY broth	20 g/L dextrine from potato starch, 10 g/L polypeptone, 5 g/L yeast extract, 5 g/L KH_2PO_4, 0.5 g/L $MgSO_4 \cdot H_2O$.		Sigma Aldrich, St. Louis, MO.
DPY agar	20 g/L dextrine from potato starch, 10 g/L polypeptone, 5 g/L yeast extract, 5 g/L KH_2PO_4, 0.5 g/L $MgSO_4 \cdot H_2O$, 25 g/L agar.		Sigma Aldrich, St. Louis, MO.
YPD	10 g/L Yeast extrac, 20 g/L Bacto peptone, 20 g/L D (+)-Glucose.		Difco 212759; Difco 211677; Merck 1.08337.

SDM	6.7 g/L Yeast nitrogen base without amino, 1.92 g/L Yeast dropout supplements without uracil, 20 g/L D-glucose, 20 g/L selective agar.	Sigma Y0626, Sigma Y1501, Merck 1.08337, Invitrogen 30391049.
LB	10 g/L Tryptone, 5 g/L Yeast extract, 5 g/L Sodium Chloride, 0.02 g/L Thymine.	oxoid LP0042; difco 212750; baker 0278; sigma T-0376.
LA	10 g/L Tryptone, 5 g/L Yeast extract, 5 g/L Sodium Chloride, 0.02 g/L Thymine, 15 g/L Agar Bacteriological.	oxoid LP0042; difco 212750; baker 0278; sigma T-0376; oxoid L11.

Table S9. Primers used in this study

Primer name	Sequence (5'-3')	Product size	Ta	Description
PKS RNA-1F	taatgccaactttgtacaaaaaagcaggctATGGCTCAATCACTGCAAGTCTATCTTTTTGG	3030 bp	60°C	*XLNpks* cloning; lower case sequence is homologous to *pEYA2* vector
PKS RNA-1R	TGCCATCTGCATGCAGGTAG			
PKS RNA-2F	TCGCAGTCACCGGTTCAAATC	2458 bp	60°C	
PKS RNA-2R	TCAGCCACTGCAATTGCTGC			
PKS RNA-3F	TGACGGTGATAAGATCGTGG	1944 bp	60°C	
PKS RNA-3R	taatgccaactttgtacaagaaagctgggtTCAAGCCAGAGCCTTCGCAATAAAAGCGCCCAC			
Padh-ChlaerFAS1-Fw	tttcaacacaagatcccaaagtcaaaATGTCTTCCTACCTTGATGTAGAGTATCTTG	2582 bp	60°C	*XLNfas1* cloning; lower case sequence is homologous to *pTYGSade* vector
FASChlaer1-1R	AGCCATCATACGGCTTCCAAG			
FASChlaer1-2F	ATTGTCCTGATTGCCGGTAG	2275 bp	60°C	
FASChlaer1-2R	TGACCGGATTTCCATGGCATG			
FASChlaer1-3F	AGGACCAGAACTTATCGGCG	1558 bp	60°C	
Tadh-ChlaerFAS1-Rv	ttcattctatgcgttatgaacatgttccctCTAGTAGTCCAAGCCTTCCAGCGCCCGACT			
Pgpd-ChlaerFAS2-Fw	aacagctaccccgcttgagcagacatcaccATGCGTGGAGACGCTAGTAAATCCAGAGATC	2752 bp	60°C	*XLNfas2* cloning; lower case sequence is homologous to *pTYGSade* vector
FASChlaer2-1R	ATCCAATTCGCAGCGTGGAG			
FASChlaer2-2F	ATCTCCTGACGCTATTGACG	1406 bp	60°C	
FASChlaer2-2R	ACTCTCAGTAGTCGGACGAG			
FASChlaer2-3F	TGTACAAGGATCAGTACCTCG	1991 bp	60°C	
Tgpd-ChlaerFAS2-Rv	acgacaatgtccatatcatcaatcatgaccCTAATAGCTTCTATCACCCTTCCCATGCAA			

vdtA_f1_F	actttgtacaaaaaagcaggctccgcATGGCGCAAAAGCTTCGT	2626 bp	60°C	*vdtA* cloning; lower case sequence is homologous to *pEYA2* vector
vdtA_f1_R	GGTCCTCTACTAGAACTGAGCTA			
vdtA_f2_F	GAACAACACTATACCTCCCCATC	2648 bp	60°C	
vdtA_f2_R	CCACATCACCCTTCCACTGA			
vdtA_f3_F	GTTCGTTAATCATGGGTGGGA	2392 bp	60°C	
vdtA_f3_R	tcggcgcgcctgtttaaactgcggccCTAGCCCTTATAACTGC			
H2B_Chlaer_F	TCTCCAACCGTGCTATGTC	gDNA: 290 bp; cDNA: 193 bp	60°C	Gene expression checking
H2B_Chlaer_R	ACAGCCTTGGTACCTTCG			
H2B_Aspory_F	GCTGCTGCCTCTGGTGAC	gDNA: 532 bp; cDNA: 381 bp	60°C	Gene expression checking
H2B_Aspory_R	GCTGCTGCCTCTGGTGAC			
PamyB-Fw	ACCGACAACATCACATCAAGC	*XLNpks* whole gene sequencing after cloning		
xln1seq-2F	TGACCTCGATCTCAGATTGG			
xln1seq-3F	TGGATGGAGACCAACTCTGC			
xln1seq-4F	TGCATTTGAGCCTACGGAATG			
xln1seq-5F	TGTCCTAACAGACTCCGATAC			
xln1seq-6F	TGCCGTTGTAAAGGCCATTG			
xln1seq-7F	TGTCCTTACGCAAACAGCTG			
xln1seq-8F	TGGATCCACCCTCTGATGCTG			
xln1seq-9F	TGAAATGCCACTACGACGAC			
xln1seq-1R	AGAGGCTGCATCAGAATCTC			
xln1seq-3R	TGCAGTAATGATGACCACGTG			
xln1seq-5R	TGAAGTCCGACTCTGCAATG			
xln1seq-7R	AGGGCTGAGTCGATCAACTC			
TamyB-Rev	ACTCACTGTCCAATGCCAG			
Padh-Fw	CCTAACTCCACCGCAACCTC			

fas1seq-1R	TGGATGCTACAACGTTGACC	*XLNfas1* whole gene sequencing after cloning
fas1seq-2F	AGGAGACTCGCGGCTATATG	
fas1seq-2R	TCACATCCTGTGATCGGAAG	
fas1seq-3F	AGTCCTCATCTTAAAGGCGC	
fas1seq-3R	TGCGAATCTTTCCGTACGCC	
fas1seq-4F	TGATTGACGGGCTGACTATTG	
fas1seq-4R	TGGAGCAACTCAGTTTCTGC	
fas1seq-5F	AGCGGTGGATAGATTCTTCC	
fas1seq-5R	TCCTGTACTCCATCTCCTCG	
fas1seq-6F	TAAGGCCATCAAGGACTCTC	
fas1seq-6R	AGGAGAACCGTATCGAGTCAG	
fas1seq-7F	TCATGACCCAGGTCACTTAC	
fas1seq-7R	TGCTGGTTGCGCGAATTGAG	
fas1seq-8F	TCCAACTACCTAGCCATGAC	
Tadh-Rv	CGATGGAATCTCATAATACTC	
Pgpd-Fw	CGAGCTTTCCCACTTCATCG	*XLNfas2* whole gene sequencing after cloning
fas2seq-1R	TACTGTGGAAGCTTGCTGAG	
fas2seq-2F	TCTAGGCAGTCTGCAGTCTTG	
fas2seq-2R	TGCGCCTGTGACTAGAACATC	
fas2seq-3F	ACCTAGATTGGTAAGAGGCC	
fas2seq-3R	TCATCACGCCTTTAGACTCG	
fas2seq-4F	TATCTCACCGTATGTGGAGC	
fas2seq-4R	AAAGCCTGTCGGCAGTTGAC	
fas2seq-5F	ATCCGACAGGTCGGAAATTC	
fas2seq-5R	ACTCCTCTGAATCTGTTGAGC	
fas2seq-6F	ACTCAACATAGACTACCGTCG	
fas2seq-6R	AGGATCCATAAGTACTGCCG	
fas2seq-7F	ACGCAACGTAAGAGACTTGC	
Tgpd-Rv	CTCTTCATTTCTTTCATTATC	

pEYA2-Fw	TCGAACTGGATCTCAACAGCG	*vdtA* whole gene sequencing after cloning
vdtA_seq_F1	TGCAGGATGTACTCCTTCG	
vdtA_f2_F	GAACAACACTATACCTCCCCATC	
vdtA_seq_F2	ATGACAGCAGCGTAGTCATG	
vdtA_f3_F	GTTCGTTAATCATGGGTGGGA	
vdtA_seq_F3	ATAGACTCGCTGCTGTCCTTG	
vdtA_seq_R1	TCCGTCTTCCAGAGTCACTG	
vdtA_seq_R2	ACACGTAGTTGATTCGGCCTG	
vdtA_seq_R3	TGCAAGCTGCATGCACGTATTG	
vdtA_seq_R4	AAGCCGTGACTCGCAGTAATTG	
vdtA_seq_R5	ATTGCTTTGGCGCGTCTGATAC	
vdtA_seq_R6	TAAGCGAGAATGCCACCG	
pEYA2-Rev	ACAGTACTGCGATGAGTGGC	
